# Hypercapnia enhances airway smooth muscle contractility via STIM1-dependent Ca^2+^ signaling

**DOI:** 10.1172/jci.insight.202475

**Published:** 2026-07-16

**Authors:** Masahiko Shigemura, Vitalii Kryvenko, Jennifer A. Pacheco, Megan J. Puckelwartz, Milos Aleksic, Natalia D. Magnani, Emma Thompson, Francisco Javier Martin-Romero, Eoin P. Cummins, Werner Seeger, Andreas Bräuninger, Lynn C. Welch, G.R. Scott Budinger, Emilia Lecuona, Laura A. Dada, Ankit Bharat, István Vadász, Murali Prakriya, Jacob I. Sznajder

**Affiliations:** 1 Division of Thoracic Surgery, Department of Surgery, Feinberg School of Medicine, Northwestern University, Chicago, Illinois, USA.; 2 Laboratory of Homeostatic Physiology, Department of Physiology and Cell Biology, Graduate School of Medicine, Kobe University, Kobe, Japan.; 3 Department of Internal Medicine, Justus Liebig University, Universities of Giessen and Marburg Lung Center, German Center for Lung Research, Cardio-Pulmonary Institute, and Institute for Lung Health, Giessen, Germany.; 4 Center for Genetic Medicine,; 5 Department of Pharmacology, and; 6 Department of Neuroscience, Feinberg School of Medicine, Northwestern University, Chicago, Illinois, USA.; 7 Faculty of Pharmacy and Biochemistry, University of Buenos Aires, Buenos Aires, Argentina.; 8 Department of Human Genetics, University of Chicago, Chicago, Illinois, USA.; 9 Department of Biochemistry and Molecular Biology, Universidad de Extremadura, Badajoz, Spain.; 10 University College Dublin School of Medicine, UCD Conway Institute of Biomolecular and Biomedical Research, Dublin, Ireland.; 11 Institute of Pathology, Justus Liebig University, Giessen, Germany.; 12 Division of Pulmonary and Critical Care Medicine, Department of Medicine, Feinberg School of Medicine, Northwestern University, Chicago, Illinois, USA.

**Keywords:** Cell biology, Muscle biology, Pulmonology, COPD, Calcium signaling, Cell stress

## Abstract

Hypercapnia, elevated carbon dioxide (CO_2_), is common in advanced chronic obstructive pulmonary disease (COPD) and predicts poor clinical outcomes. Traditionally considered a consequence of disease severity, hypercapnia may drive disease progression by promoting airway dysfunction. Here, we show that hypercapnia acts as an active stressor, driving airway smooth muscle (ASM) constriction through a stromal interaction molecule 1–dependent (STIM1-dependent) pathway. Hypercapnia rapidly activates ERK, triggering sarcoplasmic reticulum calcium (Ca^2+^) release via phosphorylation of the inositol 1,4,5-trisphosphate receptor. ERK also induces nuclear translocation of the transcription factor c-Fos, enhancing *STIM1* transcription. These responses were observed under both supraphysiological (~120 mmHg) and clinically relevant (50–60 mmHg) hypercapnia. Increased STIM1 abundance sustains store-operated Ca^2+^ entry (SOCE), amplifying ASM signaling. In mice, hypercapnia increased ASM and airway contractility in a STIM1-dependent manner. Human genetic analyses revealed noncoding *STIM1* variants associated with reduced lung expression that were enriched in patients with COPD. These variants correlated with lower airway resistance under normocapnia; however, this benefit was lost during hypercapnia, indicating a potential gene-environment interaction. Together, our findings position STIM1 as a key mechanistic node linking hypercapnia to Ca^2+^ dysregulation and airway obstruction, defining a CO_2_/ERK/STIM1/SOCE axis with translational relevance to chronic lung disease.

## Abstract 2

Elevated carbon dioxide levels can worsen airway narrowing, revealing how genetic variation and environmental stress interact to influence chronic lung disease.

## Graphical abstract

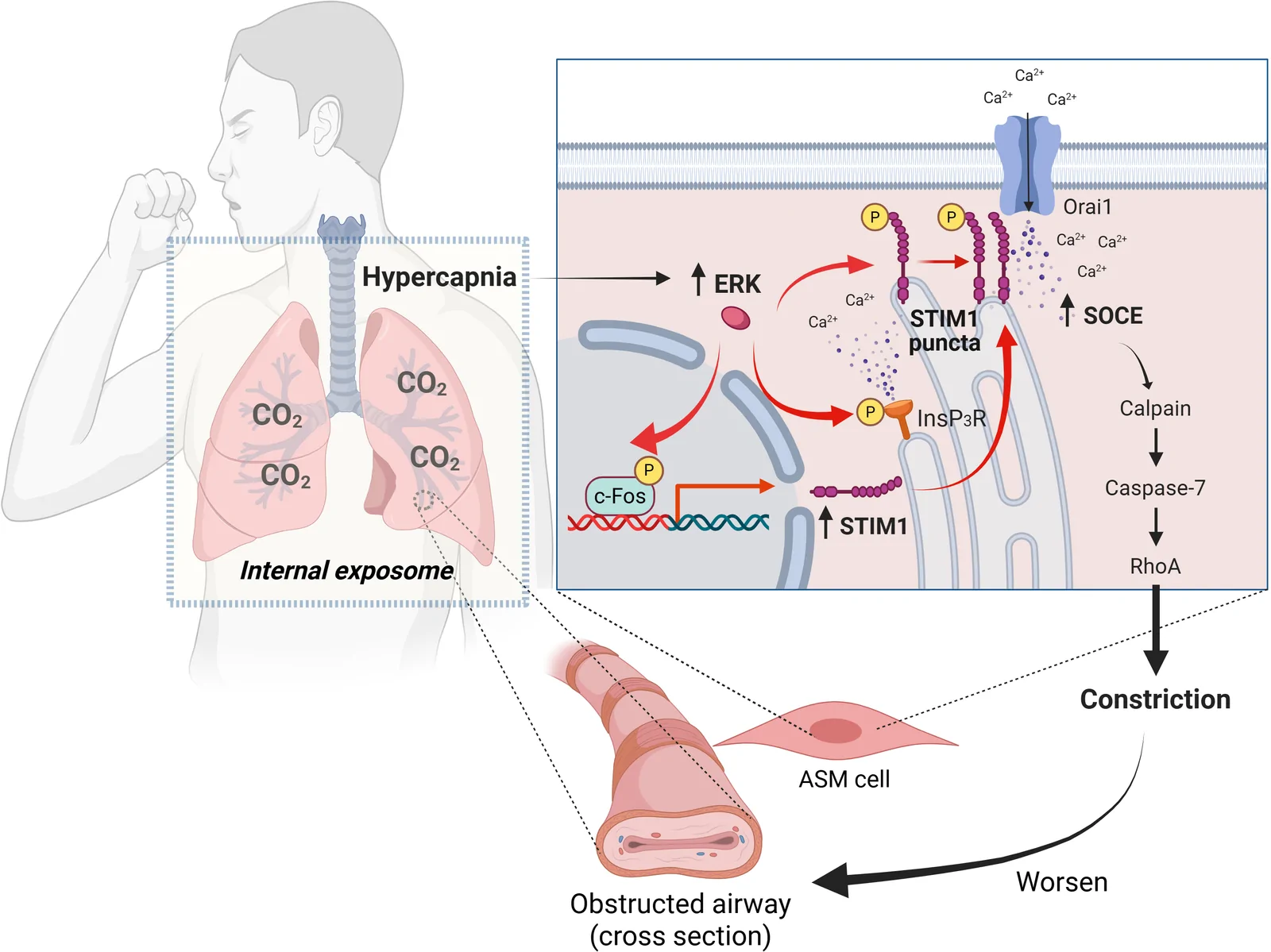

## Introduction

Cells possess evolutionarily conserved pathways that sense and adapt to changes in gaseous molecules, including oxygen and carbon dioxide (CO_2_) (1). Although considered merely a metabolic by-product, accumulating evidence indicates that elevated CO_2_ functions as a signaling molecule (2), eliciting maladaptive cellular responses in the lung (3–8) and other organ systems (9, 10).

Tight regulation of intracellular calcium (Ca^2+^) dynamics is fundamental for cellular functions, including gene transcription, metabolism, and contractility (11, 12). We previously reported that elevated CO_2_ increases intracellular Ca^2+^ levels ([Ca^2+^]_i_) in airway smooth muscle (ASM) cells, activating Ca^2+^-dependent contractile signaling that promotes ASM constriction and airway hyperresponsiveness (5). In mouse models exposed to house dust mites, Ca^2+^ influx drives ASM transcriptional and metabolic reprogramming, contributing to airway remodeling and hyperresponsiveness (13). More recently, we demonstrated that hypercapnia promotes hypertrophic remodeling of ASM in mouse lungs and human precision-cut lung slices (PCLSs) (8).

Chronic obstructive pulmonary disease (COPD), a leading global cause of morbidity and mortality, is characterized by persistent airflow limitation and progressive respiratory decline (14). Hypercapnia, defined as an elevation in arterial CO_2_ tension, is common in advanced COPD and correlates with poor clinical outcomes (15). Clinical observations indicate that COPD patients with chronic hypercapnia exhibit increased airway resistance, which improves with noninvasive ventilation aimed at reducing CO_2_ levels (5). Consistently, clinical trials have demonstrated that noninvasive ventilation improves survival and quality of life in hypercapnic COPD patients (16, 17).

While these observations establish a strong association between hypercapnia and airway dysfunction, how elevated CO_2_ engages Ca^2+^ entry pathways upstream of sustained ASM contractility remains poorly defined. Here, we show that hypercapnia acts as a biologically active stressor, driving ASM constriction and airway narrowing via a stromal interaction molecule 1–dependent (STIM1-dependent) mechanism. Elevated CO_2_ elicits a triphasic [Ca^2+^]_i_ response in ASM cells, characterized by (a) an initial transient decline, (b) Ca^2+^ release from the sarcoplasmic reticulum (SR), and (c) activation of store-operated Ca^2+^ entry (SOCE). Elevated CO_2_ rapidly activates ERK in ASM cells, initiating SR Ca^2+^ release via phosphorylation of the inositol 1,4,5-trisphosphate receptor (InsP_3_R). In parallel, ERK promotes STIM1 phosphorylation, facilitating its translocation in the context of ERK-mediated SR Ca^2+^ depletion. ERK also promotes nuclear translocation of the transcription factor c-Fos, enhancing *STIM1* transcription and thereby reinforcing SOCE during hypercapnic exposure. Extending these mechanistic observations, clinically relevant chronic hypercapnia activates the ERK/c-Fos/STIM1 axis, eliciting temporally dynamic regulation of STIM1 abundance during prolonged stimulation. This CO_2_-sensing pathway sustains Ca^2+^ entry and amplifies contractile signaling. Complementing these mechanistic studies, human genetic analyses support the notion that STIM1 abundance modulates airway contractility during hypercapnia. Noncoding *STIM1* variants associated with reduced lung expression were enriched in COPD patients and correlated with lower airway resistance under normocapnic conditions. However, this beneficial effect was lost during hypercapnia, suggesting a gene-environment interaction in which elevated CO_2_ may override genetic resilience and exacerbates airway obstruction.

Together, our findings position ERK as an upstream integrator of hypercapnia-responsive signaling that coordinates Ca^2+^ release, transcriptional remodeling, and sustained SOCE. Within this pathway, STIM1 functions as a critical mechanistic node required for amplification of Ca^2+^ entry and ASM contractility under elevated CO_2_ stress. These data provide a mechanistic framework for understanding how hypercapnia contributes to airway obstruction in chronic airway disease.

## Results

### Hypercapnia elicits a triphasic [Ca^2+^]_i_ response in ASM cells.

**Figure 1:**
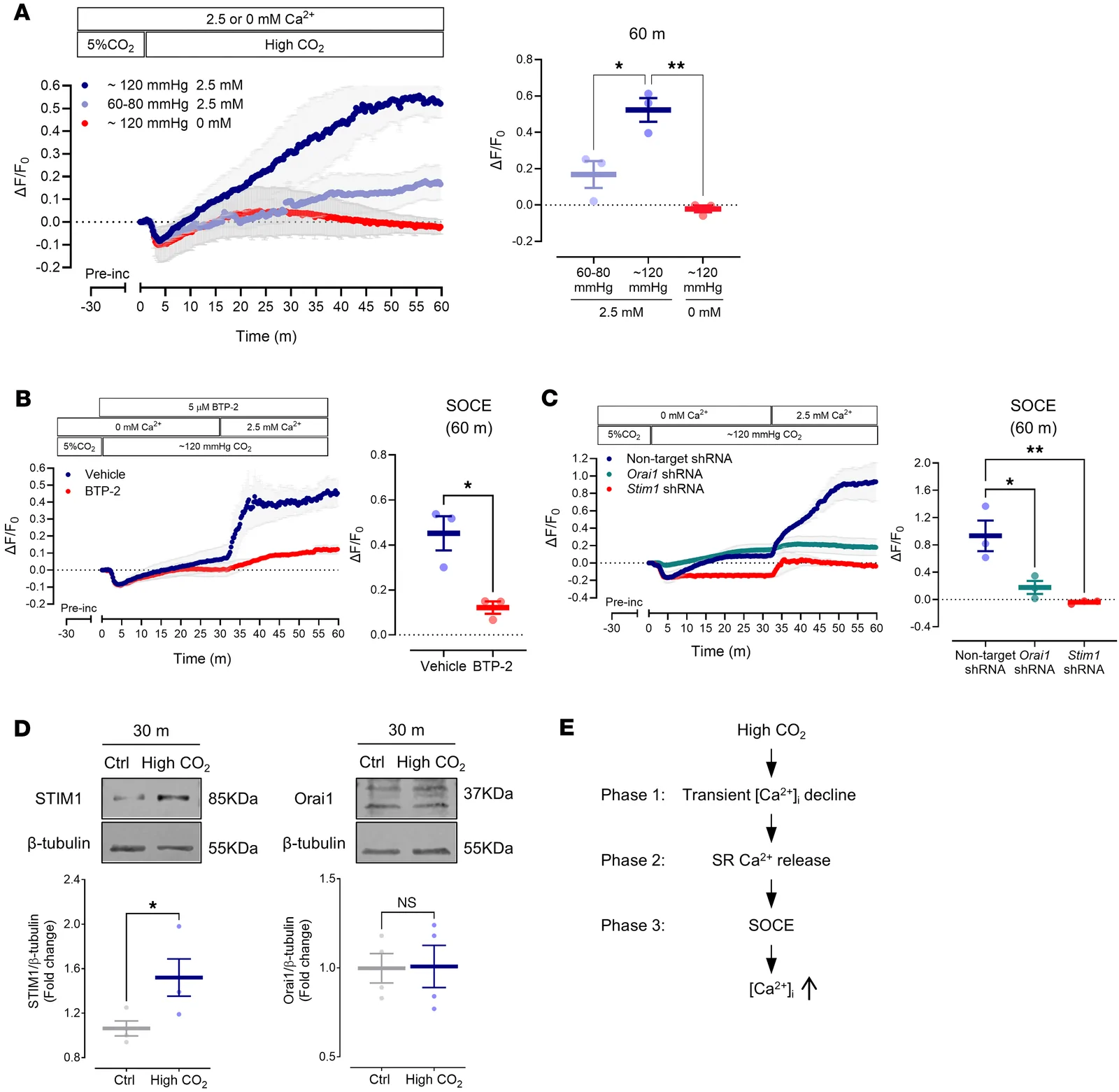
High CO_2_ elicits a triphasic Ca^2+^ response in ASM cells. (**A**–**C**) Intracellular Ca^2+^ dynamics were assessed in cultured mouse ASM cells expressing GCaMP6 (*n* = 3 biological replicates). (**A**) [Ca^2+^]_i_ traces (left) and quantification of Δ*F*/*F*_0_ at 60 minutes (right) in the presence or absence of extracellular Ca^2+^. Cells were preincubated for 30 minutes with medium equilibrated to 5% CO_2_ (30–40 mmHg, pH 7.4), then exposed to high CO_2_ (60–80 or ~120 mmHg, pH 7.4) for 60 minutes. (**B** and **C**) Ca^2+^ influx in ASM cells treated with DMSO (vehicle) or 5 μM BTP-2 (**B**), or transfected with nontarget, *Stim1*, or *Orai1* shRNA (**C**). Left: [Ca^2+^]_i_ traces. Right: Quantification of Δ*F*/*F*_0_ at 60 minutes. Cells were preincubated in 5% CO_2_ medium for 30 minutes, exposed to high-CO_2_ medium for 30 minutes in the absence of extracellular Ca^2+^, then switched to 2.5 mM Ca^2+^ for an additional 30 minutes. (**D**) Representative Western blot (top) and quantification (bottom) of STIM1 and Orai1 in ASM cells exposed to 5% CO_2_ (Ctrl; 30–40 mmHg, pH 7.4) or high CO_2_ (~120 mmHg, pH 7.4) for 30 minutes (*n* = 4 biological replicates). (**E**) Schematic illustrating the proposed triphasic [Ca^2+^]_i_ response in hypercapnic ASM cells, characterized by an initial transient decline, SR Ca^2+^ release, and SOCE activation. Data are presented as means ± SEM. Statistical analysis was performed by unpaired 2-tailed *t* test (**B** and **D**) or 1-way ANOVA with Tukey’s post hoc test (**A** and **C**). *P < 0.05, **P < 0.01. Pre-inc, preincubation; SR, sarcoplasmic reticulum; SOCE, store-operated Ca^2+^ entry.

To investigate how hypercapnia regulates intracellular Ca^2+^ dynamics in ASM, we exposed primary cultured mouse ASM cells to high CO_2_ and monitored single-cell [Ca^2+^]_i_ using the genetically encoded calcium indicator GCaMP6. Under buffered hypercapnia (60–120 mmHg CO_2_, extracellular pH 7.4), [Ca^2+^]_i_ exhibited a multiphasic response characterized by an initial transient decrease peaking at approximately 5 minutes, followed by spatially localized elevations (Ca^2+^ compartmentalization; Supplemental Video 1; supplemental material available online with this article; https://doi.org/10.1172/jci.insight.202475DS1) that persisted throughout CO_2_ exposure (Figure 1A). The sustained phase of the response was dependent on both CO_2_ concentration and exposure duration (Figure 1A).

When cells were exposed to high CO_2_ in Ca^2+^-free extracellular solution, the initial [Ca^2+^]_i_ response, comprising a transient decline following a rapid elevation, was preserved; however, the sustained increase observed in the presence of extracellular Ca^2+^ was abolished (Figure 1A). These findings indicate that extracellular Ca^2+^ is required for the sustained phase of the response. Consistent with prior reports that hypercapnia stimulates InsP_3_R-mediated Ca^2+^ release from the SR (18, 19), administration of 2-APB, an inhibitor of InsP_3_R, eliminated the recovery of [Ca^2+^]_i_ following its initial decline (Supplemental Figure 1). This result suggests that high-CO_2_-induced SR Ca^2+^ release underlies the secondary rise in [Ca^2+^]_i_. In contrast, ryanodine had no effect, indicating that ryanodine receptors are not involved in this process.

Because buffered hypercapnia is known to cause transient intracellular acidosis in various cell types (20, 21), we next examined whether intracellular pH changes contribute to the early [Ca^2+^]_i_ decline. Exposure to high CO_2_ at extracellular pH 7.2 (mimicking respiratory acidosis) caused a sustained decrease in [Ca^2+^]_i_ (Supplemental Figure 2A), coinciding with persistent intracellular acidosis throughout CO_2_ exposure (Supplemental Figure 2B). Similarly, transient intracellular acidification was observed under buffered hypercapnia, coinciding temporally with the initial [Ca^2+^]_i_ decrease. These findings suggest that the early [Ca^2+^]_i_ decline in response to hypercapnia is likely associated with intracellular acidification.

In many cell types, including smooth muscle cells, SOCE represents a primary mechanism of sustained Ca^2+^ influx (11). SOCE is mediated by Ca^2+^ release–activated calcium (CRAC) channels, including Orai1, which are activated when STIM1 senses SR Ca^2+^ depletion and translocates to SR–plasma membrane junctions (11). Increasing extracellular Ca^2+^ to 2.5 mM during hypercapnia enhanced the sustained [Ca^2+^]_i_ elevation, which was abolished by the SOCE inhibitor BTP-2 in mouse ASM cells (Figure 1B). ASM cells stably expressing shRNAs targeting *Stim1* or *Orai1* also showed significant inhibition of high-CO_2_-induced Ca^2+^ influx (Figure 1C), confirming the functional requirement of STIM1-Orai1 engagement for SOCE. Notably, hypercapnia significantly upregulated STIM1 expression, whereas Orai1 and STIM2 expression remained unchanged (Figure 1D and Supplemental Figure 3), indicating selective regulation of STIM1 without compensatory induction of related isoforms under these conditions.

Collectively, these results demonstrate that hypercapnia elicits a triphasic [Ca^2+^]_i_ response in ASM cells, comprising an initial acidification-associated decline, SR-dependent Ca^2+^ release, and sustained SOCE likely mediated through STIM1 activation (Figure 1E).

### Hypercapnia activates ERK to promote SR Ca^2+^ depletion via InsP_3_R activation.

**Figure 2:**
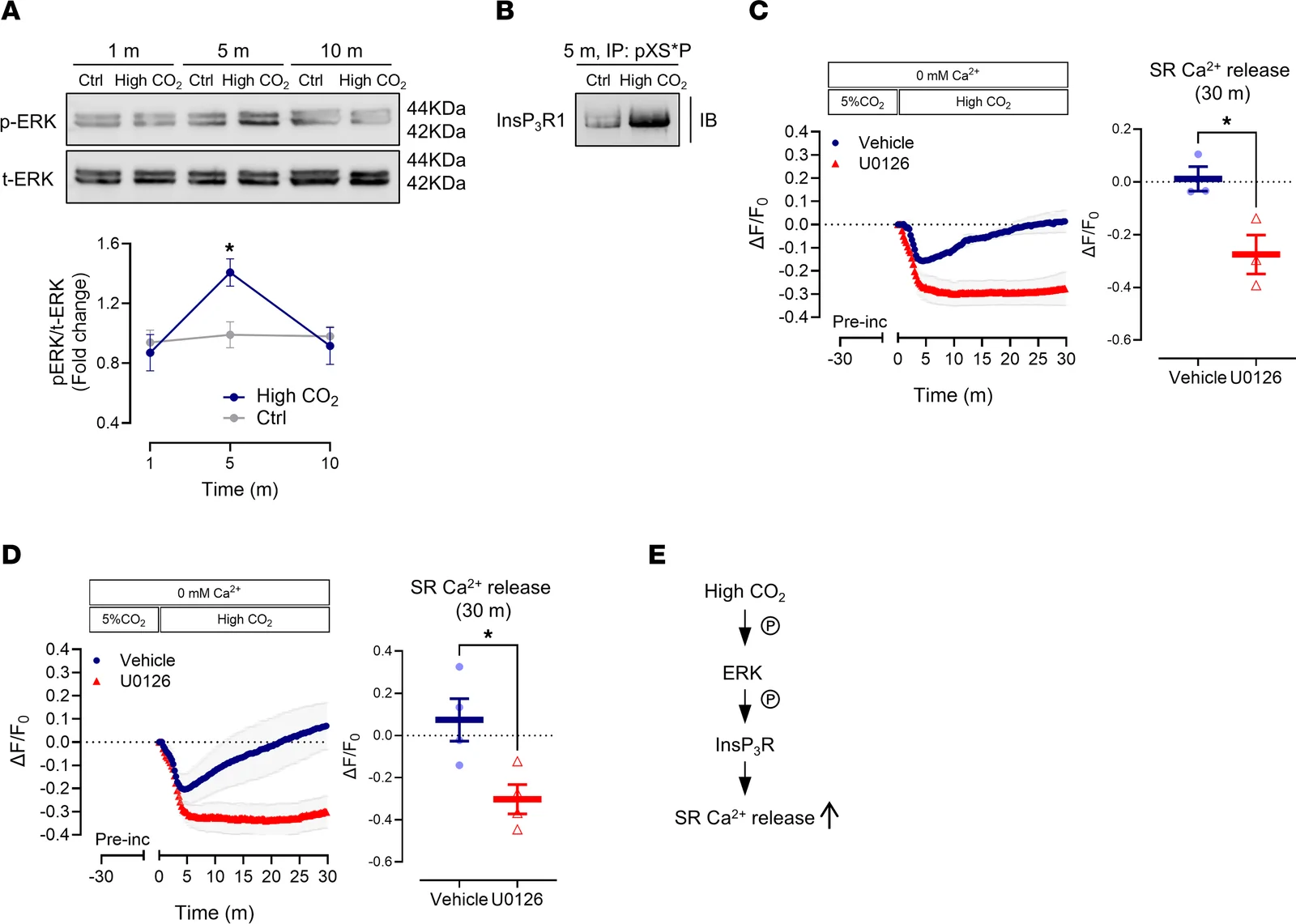
High CO_2_ activates ERK to promote SR Ca^2+^ depletion via InsP_3_R activation. Cultured ASM cells were incubated in control CO_2_ medium (Ctrl; 30–40 mmHg, pH 7.4) or exposed to buffered high CO_2_ (~120 mmHg, pH 7.4) for the indicated times. (**A**–**C**) Mouse ASM cells. (**A**) Representative Western blot (top) and quantification (bottom) of phosphorylated ERK (p-ERK) and total ERK (t-ERK) (*n* = 3 biological replicates). (**B**) Representative Western blot of proteins immunoprecipitated with anti–phospho-MAPK substrate (pXS*P) antibody from exposed cells. Blots were probed with anti-InsP_3_R1 antibody (*n* = 3 biological replicates). (**C**) [Ca^2+^]_i_ traces (left) and quantification (right) of Δ*F*/*F*_0_ in cells pretreated with DMSO (vehicle) or U0126 (10 μM, 1 hour) and continuously exposed to high CO_2_ under Ca^2+^-free conditions (*n* = 3 biological replicates). (**D**) Human ASM cells. [Ca^2+^]_i_ traces (left) and quantification (right) of Δ*F*/*F*_0_ in cells pretreated with DMSO (vehicle) or U0126 (10 μM, 1 hour) and continuously exposed to high CO_2_ under Ca^2+^-free conditions (*n* = 4 independent experimental replicates). Data are presented as means ± SEM. Statistical analysis was performed by unpaired 2-tailed *t* test (**A**, **C**, and **D**). (**E**) Schematic model illustrating ERK-mediated SR Ca2+ relesase via InsP3R activation during hypercapnia. *P < 0.05. InsP_3_R1, inositol 1,4,5-trisphosphate receptor 1; Pre-inc, preincubation; SR, sarcoplasmic reticulum.

Phosphorylation functions as a molecular switch that dynamically regulates InsP_3_R activity, controlling the rate and extent of SR Ca^2+^ store depletion (22). We previously demonstrated that hypercapnia activates ERK signaling in alveolar epithelial cells (23, 24). Consistent with these observations, exposure to high CO_2_ induced transient ERK activation in ASM cells (Figure 2A). ERK has been reported to phosphorylate InsP_3_R at optimal MAPK consensus sites (25), although the precise functional consequences of these modifications remain incompletely defined.

To determine whether hypercapnia promotes SR Ca^2+^ depletion through ERK-dependent regulation of InsP_3_R, we examined MAPK-consensus serine phosphorylation using co-immunoprecipitation with an anti–phospho-MAPK/CDK substrate antibody that recognizes phosphorylated PXSP motifs. Under high-CO_2_ conditions, immunoreactive InsP_3_R species were enriched in phospho-PXSP immunoprecipitates from mouse ASM cells (Figure 2B), consistent with increased ERK-dependent phosphorylation of InsP_3_R.

Functionally, pharmacological inhibition of ERK signaling with the MEK inhibitor U0126 prevented hypercapnia-induced SR Ca^2+^ depletion in both mouse and human ASM cells (Figure 2, C and D). Together with the data shown in Supplemental Figure 1, these findings support a model in which hypercapnia activates ERK, leading to InsP_3_R phosphorylation and enhanced SR Ca^2+^ release (Figure 2E). This pathway provides a mechanistic trigger for STIM1-dependent SOCE.

### Hypercapnia promotes STIM1 puncta formation via ERK-mediated phosphorylation.

STIM1 phosphorylation promotes its translocation to puncta-like SR–plasma membrane junctions, facilitating SOCE via Orai1 channels (26–31). ERK-dependent phosphorylation at serine residues 575, 608, and 621 has been shown to enhance this process (26–28). We hypothesized that hypercapnia promotes STIM1 accumulation at junctional SR sites through ERK-mediated phosphorylation.

**Figure 3:**
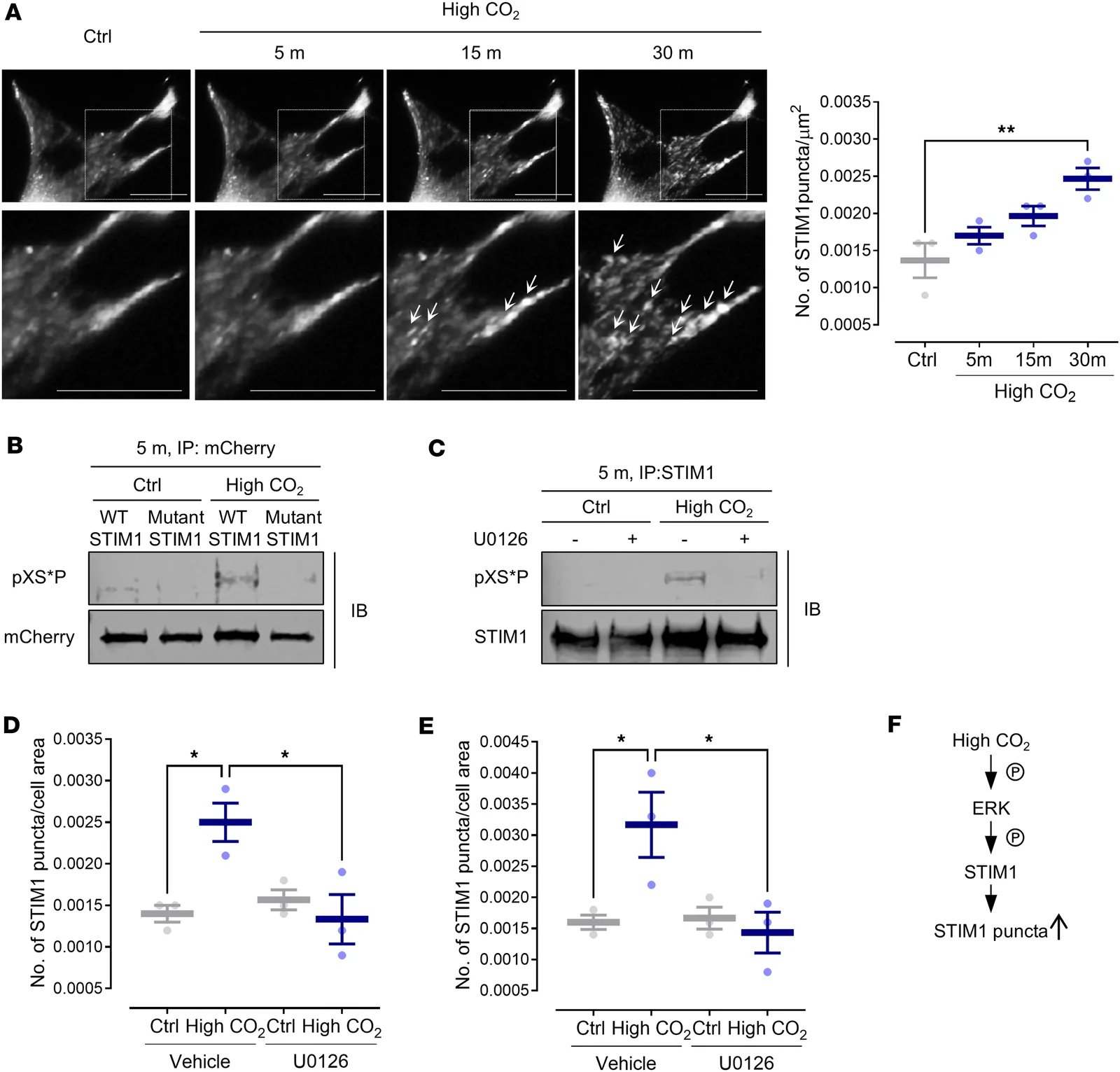
High CO_2_ promotes STIM1 puncta formation via ERK-mediated phosphorylation. Cultured ASM cells were incubated in control CO_2_ medium (Ctrl; 30–40 mmHg, pH 7.4) or exposed to buffered high CO_2_ (~120 mmHg, pH 7.4) for the indicated times. (**A**–**D**) Mouse ASM cells (*n* = 3 biological replicates). (**A**) Representative image (left) and time-course quantification (right) of mCherry-tagged STIM1 puncta formation. Cells were preincubated in Ctrl for 30 minutes, then exposed to high CO_2_. Scale bars: 50 μm. (**B**) Representative Western blot of proteins immunoprecipitated with anti-mCherry antibody from cells transfected with mCherry-tagged STIM1 wild type (WT STIM1) or STIM1^S575A/S608A/S621A^ (Mutant STIM1). Blots were probed with anti–phospho-MAPK substrate (pXS*P) and -mCherry antibodies. (**C**) Representative Western blot of proteins immunoprecipitated with anti-STIM1 antibody from cells pretreated with DMSO or U0126 (10 μM, 1 hour) and continuously exposed to high CO_2_. Blots were probed with anti–phospho-MAPK substrate (pXS*P) and -STIM1 antibodies. (**D**) Quantification of STIM1 puncta in mouse ASM cells pretreated with DMSO (Vehicle) or U0126 (10 μM, 1 hour) and continuously exposed to high CO_2_. (**E**) Quantification of STIM1 puncta in human ASM cells under the same conditions (*n* = 3 independent experimental replicates). (**F**) Schematic model illustrating ERK-mediated STIM1 phosphorylation and puncta formation during hypercapnia. Data are presented as means ± SEM. Statistical analysis was performed by 1-way ANOVA with Dunnett’s (**A**) or Tukey’s post hoc test (**D** and **E**). *P < 0.05, **P < 0.01.

To test this hypothesis, we expressed STIM1-mCherry in mouse ASM cells and monitored subcellular localization by time-lapse single-cell imaging. High CO_2_ induced redistribution of STIM1 from a diffuse SR localization in resting cells to discrete puncta at the cell periphery (Figure 3A). Immunoprecipitated STIM1 from mouse ASM cells showed increased phosphorylation detected with an anti–phospho-MAPK/CDK substrate antibody, whereas mutation of Ser575/608/621 to alanine (S575A/S608A/S621A) or treatment with U0126 abolished this effect (Figure 3, B and C). U0126 also largely prevented high-CO_2_-induced STIM1 puncta formation in both mouse and human ASM cells (Figure 3, D and E). These results suggest that hypercapnia promotes STIM1 puncta formation through ERK-mediated phosphorylation (Figure 3F), in the context of SR Ca^2+^ depletion. Our findings indicate that ERK activation is required for coordinated upstream Ca^2+^ release and downstream STIM1 redistribution in hypercapnia ASM cells.

### Hypercapnia increases STIM1 abundance via ERK/c-Fos signaling to enhance SOCE.

A large-scale transcriptomic study identified transcription factors potentially regulating hypercapnia-responsive genes, including c-Fos (32), which can rapidly increase STIM1 expression in response to stress (33, 34). c-Fos activity is regulated by ERK-mediated phosphorylation at serine 374, which stabilizes nuclear c-Fos and enhances its transcriptional function (35). Given that STIM1 abundance determines the magnitude of SOCE (36, 37), we tested whether ERK regulates STIM1 expression via c-Fos during hypercapnia.

**Figure 4:**
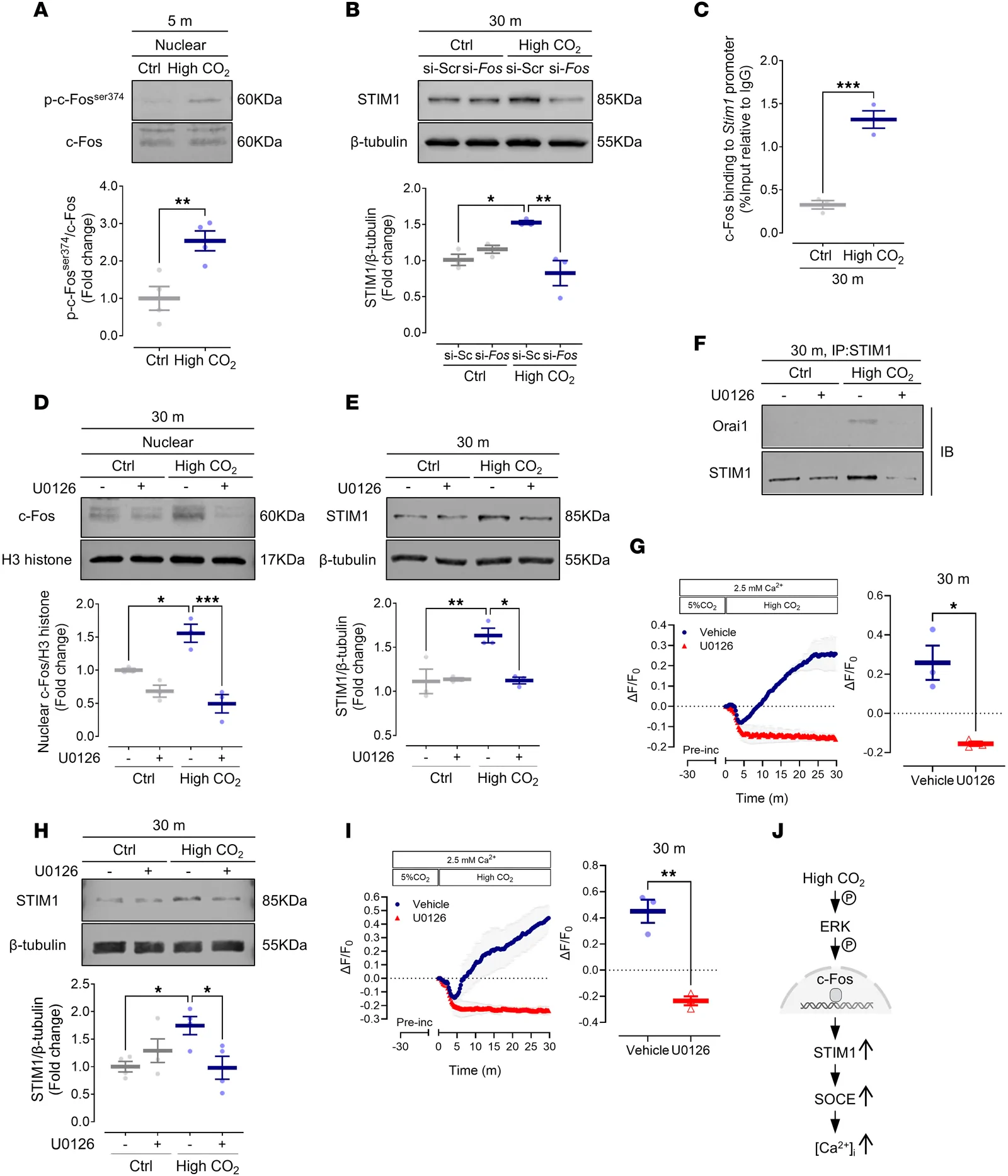
High CO_2_ increases STIM1 abundance via ERK/c-Fos signaling to enhance SOCE. Cultured ASM cells were incubated in control CO_2_ medium (Ctrl; 30–40 mmHg, pH 7.4) or exposed to buffered high CO_2_ (~120 mmHg, pH 7.4) for the indicated durations. (**A**–**G**) Mouse ASM cells. (**A** and **B**) Representative Western blot (top) and quantification (bottom) of phosphorylated c-Fos^ser374^ (**A**; *n* = 4 biological replicates), and STIM1 in cells transfected with scramble (si-Scr) or *Fos* (si-*Fos*) siRNA (**B**; *n* = 3 biological replicates). (**C**) qPCR quantification of CUT&RUN assay showing c-Fos binding to the *Stim1* promoter (*n* = 3 biological replicates). (**D**–**G**) Cells were preincubated with DMSO or U0126 (10 μM, 1 hour) and continuously exposed to the same treatment during high-CO_2_ exposure (*n* = 3 biological replicates). (**D** and **E**) Representative Western blot (top) and quantification (bottom) of c-Fos (**D**) and STIM1 (**E**). (**F**) Representative Western blot of proteins immunoprecipitated with anti-STIM1 antibody. Blots were probed with anti-STIM1 and -Orai1 antibodies. (**G**) [Ca^2+^]_i_ traces (left) and quantification (right) of Δ*F*/*F*_0_. (**H** and **I**) Human ASM cells. Representative Western blot (top) and quantification (bottom) of STIM1 (**H**; *n* = 4 independent experimental replicates) and [Ca^2+^]_i_ traces (left) and quantification (right) of Δ*F*/*F*_0_ (**I**; *n* = 3 independent experimental replicates) in cells pretreated with DMSO or U0126 (10 μM, 1 hour) and continuously exposed to the same treatment during high-CO_2_ exposure. (**J**) Schematic model illustrating ERK-dependent c-Fos activation, STIM1 upregulation, and enhanced SOCE during hypercapnia. Data are presented as means ± SEM. Statistical analysis was performed by unpaired 2-tailed *t* test (**A**, **C**, and **I**), 1-way ANOVA with Tukey’s post hoc test (**B**, **D**, **E**, and **H**), or Welch’s *t* test (**G**). *P < 0.05, **P < 0.01, ***P < 0.001. Pre-inc, preincubation.

High CO_2_ caused transient ERK activation (Figure 2A) and increased nuclear c-Fos phosphorylation (Figure 4A). Silencing *Fos* with siRNA abrogated high-CO_2_-induced STIM1 upregulation (Figure 4B). Chromatin immunoprecipitation confirmed enhanced c-Fos binding to the *Stim1* promoter under hypercapnia (Figure 4C). Pharmacological ERK inhibition with the MEK inhibitor U0126 prevented hypercapnia-induced c-Fos translocation and STIM1 protein abundance (Figure 4, D and E). We next examined whether hypercapnia-induced STIM1 activation facilitates its interaction with CRAC channels. Co-immunoprecipitation demonstrated increased STIM1-Orai1 association under hypercapnia, which was abolished by U0126 (Figure 4F). The ERK inhibition also attenuated high-CO_2_-induced elevations in [Ca^2+^]_i_ (Figure 4G). These findings that hypercapnia activates the ERK/STIM1 pathway, increasing [Ca^2+^]_i_, were recapitulated in human cultured ASM cells (Figure 4, H and I). Together, our data indicate that ERK-dependent c-Fos activation increases STIM1 abundance and promotes STIM1-Orai1 interaction, sustaining SOCE during hypercapnia (Figure 4J).

### Hypercapnia induces ERK-dependent STIM1 signaling required for ASM contraction.

**Figure 5:**
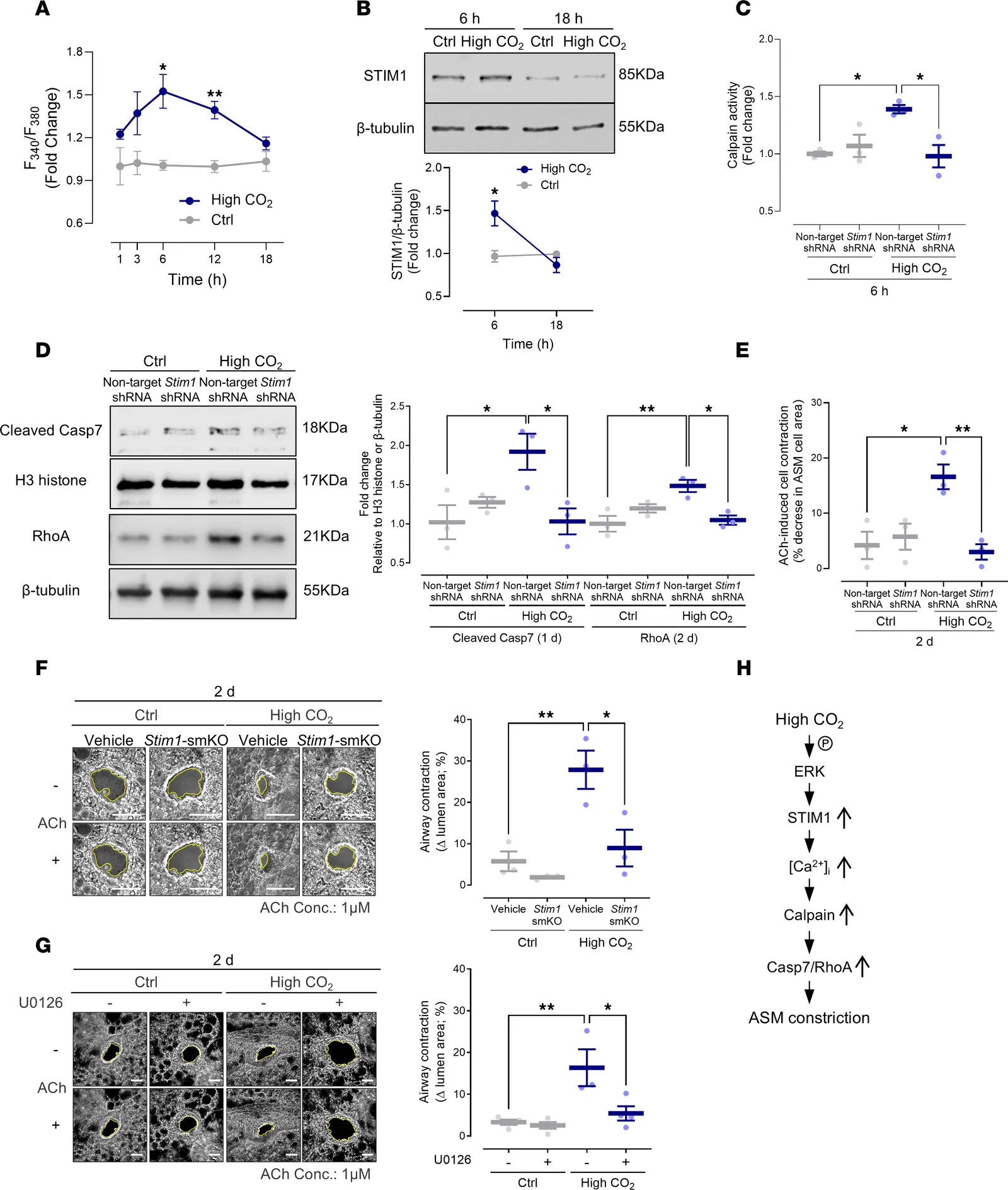
High CO_2_ induces ERK-dependent STIM1 signaling required for ASM contraction. Cultured mouse ASM cells or PCLSs were incubated in control CO_2_ medium (Ctrl; 30–40 mmHg, pH 7.4) or exposed to buffered high CO_2_ (~120 mmHg, pH 7.4) for the indicated times. (**A** and **B**) ASM cells. (**A**) [Ca^2+^]_i_ measured using Fura-2 AM (*n* = 3 biological replicates). (**B**) Representative Western blot (top) and quantification (bottom) of STIM1 (*n* = 3 biological replicates). (**C**–**E**) ASM cells expressing non-targeting or *Stim1* shRNA. (**C**) Calpain activity (*n* = 3 biological replicates). (**D**) Representative Western blots (left) and quantification (right) of nuclear cleaved caspase-7 (Casp7) and RhoA (*n* = 3 biological replicates). (**E**) Quantification of contraction induced by acetylcholine (ACh) (*n* = 3 biological replicates). (**F** and **G**) Representative images (left) and quantification (right) of ACh-induced airway contraction in PCLSs. (**F**) PCLSs obtained from *Stim1*-smKO (*n* = 3 biological replicates). Scale bars: 100 μm. (**G**) PCLSs obtained from C57BL/6J wild-type mice were pretreated with DMSO or 10 μM U0126 for 1 hour, followed by continuous exposure to Ctrl or high CO_2_ (*n* = 3–4 biological replicates). Scale bars: 200 μm. (**H**) Schematic illustrating hypercapnia-induced ASM contractility mediated via ERK/STIM1-dependent Ca^2+^ signaling pathways, thereby driving contractile responses. Data are presented as means ± SEM. Statistical analysis was performed by unpaired *t* test. (**A** and **B**) or 1-way ANOVA with Tukey’s post hoc test (**C**–**G**). *P < 0.05, **P < 0.01.

To examine the dynamics of hypercapnia-responsive signaling, we assessed the effects of elevated CO_2_ (~120 mmHg, extracellular pH 7.4) for up to 48 hours in cultured mouse ASM cells. This approach allows dissection of early mechanistic events triggered by elevated CO_2_ (3, 5, 8). Both [Ca^2+^]_i_ and STIM1 protein abundance remained elevated for up to 6 hours of high-CO_2_ exposure (Figure 5, A and B), supporting sustained STIM1-dependent Ca^2+^ signaling.

To determine the functional role of STIM1 in hypercapnia-induced ASM constriction, we examined hypercapnia-responsive signaling and acetylcholine-induced contractility in ASM cells expressing either non-targeting or *Stim1*-targeting shRNA. Consistent with prior findings (5), high CO_2_ increased calpain activation, nuclear caspase-7 cleavage, RhoA expression, and acetylcholine-induced contraction in cells expressing non-targeting shRNA, whereas these responses were abolished in *Stim1*-deficient cells (Figure 5, C–E).

These findings were further validated in PCLSs from C57BL/6J wild-type or smooth muscle–specific *Stim1* knockout (*Stim1*-smKO) mice. High CO_2_ enhanced acetylcholine-induced airway contraction in wild-type PCLSs, which was abrogated by smooth muscle–specific *Stim1* deletion (Figure 5F) or pharmacological ERK inhibition (Figure 5G). These small-airway PCLS data provide key functional evidence supporting the requirement for STIM1- and ERK-dependent signaling in hypercapnia-induced airway constriction. Together, these results indicate that ERK-dependent STIM1 signaling is a critical driver of hypercapnia-induced ASM contraction and airway responsiveness (Figure 5H).

Beyond contractility, hypercapnia may induce early phenotypic changes in ASM relevant to airway remodeling. We have recently reported that prolonged hypercapnia promotes ASM hypertrophy in mouse and human models (8). Here, we assessed α–smooth muscle actin (α-SMA) expression and proliferation in ASM cells under sustained hypercapnia. High CO_2_ increased α-SMA expression and impaired proliferation, effects that were prevented by *Stim1* knockdown (Supplemental Figure 4). These data suggest that STIM1 contributes not only to acute Ca^2+^-dependent contractile responses but also to early remodeling-associated ASM phenotypic changes.

Collectively, these findings reveal a STIM1-dependent mechanistic link between elevated CO_2_ stress and airway dysfunction, suggesting that STIM1 plays a role in chronic airway disease.

### Hypercapnia drives a biphasic STIM1 response across acute and chronic phases.

To extend our mechanistic findings to clinically relevant conditions, we examined human ASM cells exposed to acute and prolonged hypercapnia (50–60 mmHg CO_2_, extracellular pH 7.4) and compared these responses with supraphysiological hypercapnia (~120 mmHg CO_2_, extracellular pH 7.4).

**Figure 6:**
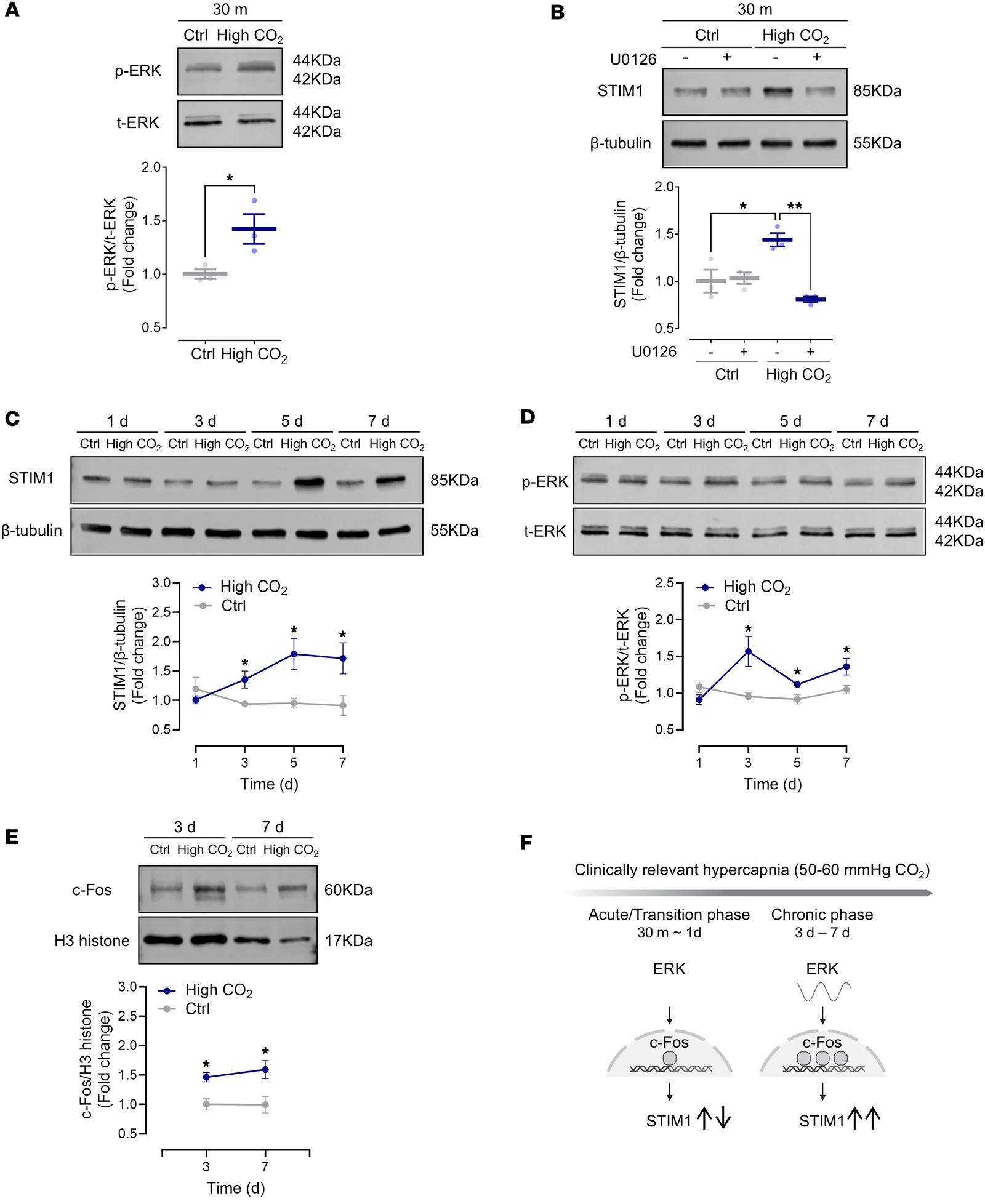
Hypercapnia drives a biphasic STIM1 response across acute and chronic phases. Cultured human ASM cells were incubated in control CO_2_ medium (Ctrl; 30–40 mmHg, pH 7.4) or exposed to buffered high CO_2_ (50–60 mmHg, pH 7.4) for the indicated durations. (**A** and **B**) Acute exposure phase (30 minutes). Representative Western blot (top) and quantification (bottom) of phosphorylated ERK (p-ERK; **A**), and STIM1 (**B**) in cells pretreated with DMSO or U0126 (10 μM, 1 hour) and maintained in the same treatment conditions during high-CO_2_ exposure (*n* = 3 independent experimental replicates). (**C**–**E**) Chronic exposure phase (up to 7 days). Representative Western blot (top) and quantification (bottom) of STIM1 (**C**; *n* = 4 independent experimental replicates), p-ERK (**D**; *n* = 4 independent experimental replicates), and nuclear c-Fos (**E**; *n* = 3 independent experimental replicates). (**F**) Schematic model illustrating the biphasic STIM1 response to clinically relevant hypercapnia, accompanied by temporally distinct ERK activation and persistent nuclear c-Fos localization. Data are presented as means ± SEM. Statistical analysis was performed by unpaired *t* test (**A** and **C**–**E**) or 1-way ANOVA with Tukey’s post hoc test (**B**). *P < 0.05, **P < 0.01. t-ERK, total ERK.

During the acute phase, clinically relevant hypercapnia induced activation of the ERK/c-Fos/STIM1 signaling axis (Figure 6, A and B), albeit with delayed kinetics compared with supraphysiological hypercapnia. Specifically, ERK phosphorylation was detected at 30 minutes after CO_2_ exposure (Figure 6A and Supplemental Figure 5A), whereas activation occurred within 5 minutes under 120 mmHg CO_2_ conditions (Figure 2A). These findings indicate that the magnitude of hypercapnic stress modulates signaling kinetics without altering the directionality of pathway activation.

To determine whether STIM1 regulation is sustained over time, we next performed extended time-course analyses under clinically relevant hypercapnia. STIM1 protein abundance showed an initial increase (Figure 6B) followed by a transient normalization phase at 24 hours, and subsequently a secondary sustained increase during prolonged exposure (3–7 days) (Figure 6C). This biphasic pattern was accompanied by temporally distinct ERK activation (Figure 6D) and persistent nuclear c-Fos localization (Figure 6E). We also observed a similar directional regulation of STIM1 in mouse ASM cells exposed to clinically relevant chronic hypercapnia (Supplemental Figure 5B).

Collectively, these data demonstrate that hypercapnia induces a temporally mediated STIM1 response characterized by an early induction phase, transient normalization, and delayed secondary activation through ERK/c-Fos regulation (Figure 6F). This dynamic regulation provides a mechanistic framework linking acute hypercapnic signaling to chronic ASM dysfunction.

### Gene-environment interaction between STIM1 variants and hypercapnia is associated with altered airway resistance in patients with COPD.

As a disease-relevant context for hypercapnia-induced airway contractility, prior cohort studies demonstrated that airway resistance is higher in hypercapnic than in normocapnic patients with COPD and improves following noninvasive ventilation aimed at correcting hypercapnia (5). A recent study reported a trend linking STIM1 abundance with airflow limitation in COPD lung tissue (38). To test whether our mechanistic findings extend to humans, we interrogated publicly available datasets and analyzed 2 independent COPD cohorts.

**Figure 7:**
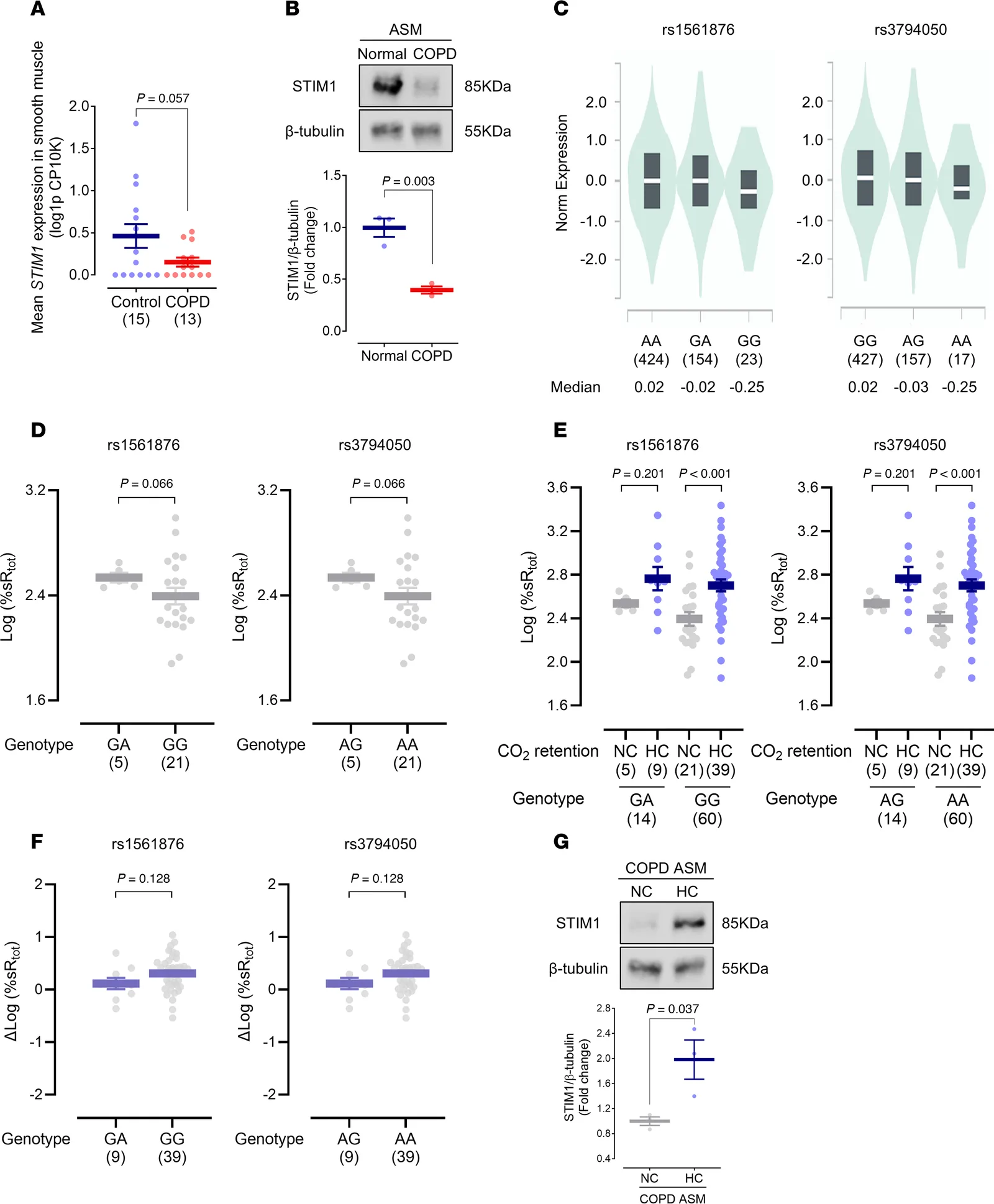
Interaction between hypercapnia and *STIM1* variants influences airway obstruction in COPD. (**A**) *STIM1* expression in smooth muscle cells from explanted COPD lungs and healthy controls, analyzed using the COPD Cell Atlas (GSE136831). Each dot represents the per-subject mean log-normalized STIM1 expression across smooth muscle cells. (**B**) Representative Western blot (top) and quantification (bottom) of STIM1 in ASM cells derived from normal or COPD lung tissue (*n* = 3 independent experimental replicates). (**C**) Genotype-specific *STIM1* expression in 601 normal lung tissues, visualized using the GTEx eQTL calculator (https://www.gtexportal.org/home/testyourown). (**D**) Log (%sRtot, percentage of total specific resistance) in 26 normocapnic patients with chronic stable COPD from cohort study 2, grouped by minor allele zygosity (homozygous vs. heterozygous). (**E**) Log (%sR_tot_) in 74 patients with chronic stable COPD from cohort study 2, grouped by CO_2_ retention status (normocapnia [NC] vs. hypercapnia [HC]) and minor allele zygosity (homozygous vs. heterozygous). Main effect of CO_2_: *P* = 0.008. (**F**) Change (Δ) in log (%sR_tot_) in each genotype. Δ log (%sR_tot_) = log (%sR_tot_) from subjects with hypercapnia – averaged log (%sR_tot_) from normocapnic heterozygotes. (**G**) Representative Western blot (top) and quantification (bottom) of STIM1 in ASM cells from COPD donor exposed to control CO_2_ medium 30–40 mmHg, pH 7.4) or buffered high CO_2_ (HC; 50–60 mmHg, pH 7.4) for 7 days (*n* = 3 independent experimental replicates). Data are presented as means ± SEM. Statistical analysis was performed by unpaired (**B**, **F**, and **G**) or Welch’s (**A** and **D**) 2-tailed *t* test and 2-way ANOVA with post hoc uncorrected Fisher’s least significant difference test (**E**). Sample sizes and *P* values are indicated in the figure unless otherwise specified.

Analysis of the COPD Cell Atlas (39) revealed that *STIM1* expression is highly context and cell type dependent, with increased expression in specific epithelial lineages, while showing reduced expression in stromal populations, including smooth muscle cells, in explanted COPD lungs compared with controls (Figure 7A and Supplemental Figure 6). STIM1 expression has been shown to be lower in ASM cells associated with a differentiated contractile phenotype compared with a synthetic-like state, supporting tight regulation of STIM1 expression in relation to ASM cell state (40, 41). Consistent with these single-cell transcriptomic data, our in vitro experiments demonstrated lower STIM1 protein abundance in COPD-derived ASM cells compared with normal ASM cells (Figure 7B), suggesting that disease-associated differences in ASM phenotype may contribute to altered STIM1 expression.

Consistent with these expression patterns, several noncoding *STIM1* variants (rs1561876, rs3750994, rs3750996, rs3794050, and rs7934581) have been associated with altered *STIM1* expression, with minor allele carriers showing reduced transcript levels in whole blood (42). Consistently, GTEx expression quantitative trait locus (eQTL) analysis identified rs1561876 and rs3794050 as loci influencing *STIM1* expression in normal lung tissue, with minor alleles trending toward reduced expression (Figure 7C). Given their near-complete linkage disequilibrium (*R*^2^ = 0.981), we focused subsequent analyses on these two variants.

In cohort study 1, a case-control study of 118 European-ancestry subjects matched for age, sex, and smoking status, we first confirmed that potential confounders (age, sex, smoking status, BMI) did not influence the prevalence of rs1561876 and rs3794050 in 828 healthy European-ancestry individuals (Supplemental Figure 7 and Supplemental Table 1). Minor allele frequencies for both rs1561876 (adjusted OR: 9.85; *P* = 7.98 × 10^–6^) and rs3794050 (adjusted OR: 12.5; *P* = 6.26 × 10^–6^) were significantly higher in COPD patients than in healthy controls (Table 1), suggesting that these variants may contribute to reduced *STIM1* expression in COPD.

In cohort study 2, we assessed the relationship between *STIM1* variants and airway resistance in European-ancestry smokers with COPD carrying the minor alleles, stratified by CO_2_ retention status (Table 2). Disease severity did not differ significantly by genotype within either normocapnic or hypercapnic subgroups; however, as expected, severity overall was higher in the hypercapnic group (Table 2 and Supplemental Table 2). Among normocapnic individuals, homozygous carriers of the minor alleles showed a trend toward lower airway resistance (Figure 7D). Two-way ANOVA revealed a significant main effect of CO_2_ retention (*F*_1,70_ = 7.488, *P* = 0.008), indicating that hypercapnia increased airway resistance (Figure 7E). In contrast, the main effect of genotype was not significant (*F*_1,70_ = 1.104, *P* = 0.297), and no significant interaction was detected (*F*_1,70_ = 0.170, *P* = 0.682). Notably, the change in airway resistance (hypercapnia – normocapnia) did not differ between genotypes, suggesting that the beneficial association of the minor alleles under normocapnia was abolished under hypercapnic conditions (Figure 7F).

In vitro, exposure of ASM cells derived from COPD lung tissue to clinically relevant hypercapnia (50–60 mmHg, extracellular pH 7.4) (5, 8) for 7 days resulted in increased STIM1 abundance (Figure 7G). This sustained effect highlights the pathological relevance of STIM1 signaling and aligns with the clinical observation that the lower airway resistance associated with minor allele carriage under normocapnia was overridden by hypercapnia.

Taken together, these findings suggest that specific *STIM1* variants may predispose individuals to altered airway contractility, potentially through modulation of gene expression, and that hypercapnia can exacerbate airway obstruction by overriding this genetic resilience, consistent with a gene-environment interaction in patients with COPD.

## Discussion

Hypercapnia is increasingly recognized not merely as a marker of disease severity, but as an active driver of lung pathology (2). In patients with COPD, hypercapnia independently predicts mortality (43), even after adjustment for other major confounders (44). Identifying individuals at risk for hypercapnia is therefore critical to mitigate its detrimental consequences and guide personalized therapeutic strategies.

Intracellular Ca^2+^ is a central mediator of stress-adaptive signaling, particularly during hypoxia (45). We and others have previously shown that elevated CO_2_ increases [Ca^2+^]_i_ in ASM and other cell types (3, 5, 19), although the underlying mechanisms remain incompletely defined. Here, we demonstrate that hypercapnia elicits a triphasic Ca^2+^ response in ASM cells, characterized by an initial transient decline, SR Ca^2+^ release, and subsequent SOCE. This distinct temporal pattern reflects a coordinated sequence in which intracellular acidification precedes SR Ca^2+^ mobilization and Ca^2+^ influx, defining a unique Ca^2+^ signaling signature of hypercapnia.

We further identify ERK-dependent pathways linking hypercapnia to sustained Ca^2+^ signaling in ASM. Hypercapnia rapidly activates ERK, which phosphorylates InsP_3_R, triggering SR Ca^2+^ mobilization and subsequent STIM1 activation. ERK also phosphorylates c-Fos, promoting its nuclear translocation and transcriptional upregulation of STIM1. Upregulated STIM1 then drives persistent SOCE and prolonged contractile signaling, a cascade that likely represents a CO_2_ stress-sensing and amplification mechanism that contributes to ASM constriction. To extend these mechanistic observations, we examined ERK/STIM1 signaling under chronic hypercapnia conditions. Extended time-course analyses revealed a biphasic pattern of STIM1 regulation, characterized by an initial increase, a transient normalization phase, and a delayed secondary increase during prolonged exposure, accompanied by sustained but temporally variable ERK activation and persistent nuclear c-Fos localization. These findings may reflect a temporal shift from acute adaptive signaling toward chronic maladaptive responses during sustained hypercapnia.

Our findings indicate that the SR serves as the primary source of elevated [Ca^2+^]_i_ in hypercapnic ASM cells through InsP_3_R-dependent Ca^2+^ release, consistent with prior evidence that elevated CO_2_ depletes SR Ca^2+^ stores (18, 19). SR Ca^2+^ depletion, whether physiologically partial or pharmacologically complete, triggers STIM1 translocation to SR–plasma membrane junctions, forming puncta that activate SOCE (46–48). In our model, hypercapnia also rapidly induces ERK-dependent STIM1 phosphorylation, and pharmacological ERK inhibition prevents STIM1 puncta formation. STIM1 phosphorylation has been shown to regulate its interaction with Orai1 and to facilitate SOCE activation (26–31). In some systems, ERK-dependent phosphorylation at S575/608/621 promotes STIM1 dissociation from microtubule plus-end-binding proteins, enabling Orai1 engagement and SOCE (26, 27), although other studies report minimal effects of alanine substitutions at these residues (49).

The transient initial decline in [Ca^2+^]_i_ observed after acute CO_2_ exposure was unexpected, but aligns with prior evidence that intracellular acidification suppresses Ca^2+^ influx through voltage-dependent and CRAC channels, thereby transiently lowering [Ca^2+^]_i_ (50–52). We found that buffered hypercapnia induced a brief intracellular acidosis coinciding with this early [Ca^2+^]_i_ decrease, suggesting activation of Ca^2+^ extrusion or intracellular buffering mechanisms during the initial phase of elevated CO_2_ stress.

Our study distinguishes between acute mechanistic signaling events and the effects of clinically relevant chronic hypercapnia. Acute exposure to higher CO_2_ (~120 mmHg) enabled temporal resolution of upstream signaling events, including ERK activation, c-Fos nuclear translocation, and STIM1 activation, thereby establishing causal relationships linking hypercapnia to downstream Ca^2+^ entry and ASM contractility. Genetic and pharmacological loss-of-function approaches confirmed that STIM1 is functionally required for both acute and sustained hypercapnia-induced ASM constriction. Importantly, chronic exposure to clinically relevant hypercapnia (50–60 mmHg CO_2_, pH 7.4, 7 days) increased STIM1 protein abundance in ASM derived from both normal and COPD lungs. These results, together with independent functional evidence from prior mouse and human PCLS studies under chronic hypercapnia (5, 8), support a model in which increased STIM1 abundance contributes to hypercapnia-induced ASM contractility. This framework provides a conceptual explanation for how sustained or repeated engagement of immediate-early signaling pathways by hypercapnia may cumulatively drive STIM1-dependent ASM dysfunction in chronic airway disease.

Several limitations of this study warrant consideration. Supraphysiological levels of hypercapnia (~120 mmHg) were employed in selected in vitro and ex vivo experiments to enable rapid activation and dissection of hypercapnia-responsive signaling pathways. Notably, lower, clinically relevant CO_2_ levels (50–60 mmHg) elicit similar downstream effects when applied over prolonged periods, consistent with prior (3, 5, 8) and current studies. Across these partial pressure of carbon dioxide (pCO_2_) ranges, hypercapnia-induced increases in STIM1 protein abundance were observed in both mouse and human ASM cells, supporting the physiological relevance of this pathway. Although this study focuses on a major STIM1/Orai1 signaling axis driving ASM contractility, contributions from other Orai isoforms or Ca^2+^ entry pathways cannot be excluded. While other stress-responsive pathways may modulate cellular responses to hypercapnia in a context-dependent manner, our data identify the CO_2_/ERK/STIM1/SOCE axis as a central and necessary pathway driving ASM contractility. Additional limitations include the retrospective design of our COPD genetic cohorts and the indirect assessment of STIM1 genotype-phenotype associations. These data should be interpreted as supportive of the mechanistic model rather than as definitive clinical evidence. The relatively small number of minor allele carriers may have limited statistical power, and *STIM1* expression was not directly measured in these cohorts. Moreover, hypercapnia may exacerbate airway dysfunction through mechanisms independent of STIM1 abundance. Nevertheless, the observed association between *STIM1* variants and airway resistance is consistent with our mechanistic findings and supports a potential gene-environment interaction model. Prospective, genotype-stratified studies will be required to confirm these associations.

Within this framework, our findings suggest that hypercapnia functions as a potent microenvironmental stressor, contributing directly to pathological airway constriction. Integrating mechanistic proof-of-principle analyses with cellular and ex vivo functional validation and human genetic data, we propose a biologically relevant pathway in which hypercapnia can override genetic resilience and exacerbate airway obstruction through STIM1-dependent signaling. The phenotypic heterogeneity of COPD (14) reflects its multifactorial etiology and diverse molecular drivers, making it an ideal context for exposome-based research (53, 54). In this context, hypercapnia can be conceptualized as a chronic, internalized component of the exposome. Sustained exposures such as cigarette smoke, a source of inhaled CO_2_ (55), may counteract the beneficial effects of *STIM1* variants and accelerate disease progression through convergent signaling pathways, as cigarette smoke extract increases STIM1 expression in ASM and other cell types (38, 56).

In summary, we identify a CO_2_-responsive, STIM1-dependent signaling axis in ASM linking an internalized stressor to contractile dysfunction and airway obstruction. These findings advance mechanistic understanding of COPD pathobiology and provide a rationale for therapeutic strategies that reduce CO_2_ retention, such as noninvasive ventilation (16, 17). The CO_2_/ERK/STIM1/SOCE axis represents a potential therapeutic target in hypercapnic COPD, supporting precision medicine approaches for disease stratification and intervention.

## Methods

Further information can be found in Supplemental Methods.

### Sex as a biological variable

Our study included both male and female mice and human models. Similar effects of hypercapnia on airway contractility were confirmed in both sexes (5), and thus sex was not considered a biological variable in our analysis.

### Study design

This study was designed to define upstream mechanisms of hypercapnia-responsive Ca^2+^ signaling in airway smooth muscle (ASM) and to test the hypothesis that hypercapnia functions as a microenvironmental stressor that activates STIM1-dependent signaling pathways, thereby enhancing airway contractility. We employed mechanistic proof-of-principle experiments using a multidisciplinary approach that integrated cellular studies and ex vivo functional validation, and human genetic analyses for translational context.

#### Mechanistic studies.

Primary mouse tracheal ASM cells were used as an initial mechanistic platform to examine the effects of elevated CO_2_ on intracellular Ca^2+^ dynamics and STIM1-dependent signaling. Key findings were subsequently validated in human bronchial ASM cells. Experimental approaches included live-cell Ca^2+^ imaging, siRNA-mediated gene silencing, and pharmacological inhibition. Building on our previous work suggesting that sustained hypercapnia increases [Ca^2+^]_i_ through altered Ca^2+^ compartmentalization (5), we used a genetically encoded Ca^2+^ indicator to assess localized and sustained Ca^2+^ signaling dynamics.

#### Functional validation.

Precision-cut lung slices (PCLSs) from wild-type or smooth muscle–specific *Stim1* knockout mice were used to determine whether hypercapnia enhances airway contractility in a STIM1-dependent manner in intact small-airway tissue.

#### Two-tailed Human genetic analyses for translational context.

In prior COPD cohort studies, hypercapnia was associated with increased airway contractility, whereas airway resistance was reduced following noninvasive ventilation aimed at lowering the partial pressure of CO_2_ in arterial blood (PaCO_2_), providing a disease-relevant context for examining ASM contractile signaling. To complement the current experimental work, we performed targeted analyses of publicly available datasets and 2 modest COPD cohorts. These analyses were not intended as definitive population-level genetic studies, but rather to provide human disease context and to assess whether noncoding *STIM1* variants associate with gene expression and airway resistance in a manner consistent with the experimentally defined mechanistic model.

Sample sizes for in vitro and ex vivo experiments were guided by pilot studies, prior work from our laboratory, and published literature, with an emphasis on reproducibility and internal consistency across independent experiments.

### Cells lines and culture

Mouse tracheal ASM cells were isolated and cultured as previously described (5). Human bronchial ASM cells (PCS-130-011, ATCC) were maintained in culture medium consisting of DMEM supplemented with 10% FBS, penicillin (100 U/mL), and streptomycin (100 μg/mL). COPD-derived bronchial ASM cells were obtained from PromoCell (C-12561, lot 499Z012.2) and cultured according to the manufacturer’s instructions using the recommended growth medium (C-22262, C-39262, PromoCell) to preserve disease-associated cellular phenotypes.

Mouse ASM cell experiments incorporated independent biological replicates derived from multiple animals. For human ASM studies, *n* denotes independent experimental replicates performed using commercially obtained human ASM cells and does not represent distinct biological donors.

All experiments were performed using cells at passages lower than 6.

### CO2 medium and CO2 exposure

For experimental conditions, initial solutions were prepared with culture medium/Ham’s F-12 medium/Tris base/MOPS base (3:1:0.25:0.25), as previously described (5, 9). For experiments with COPD-derived bronchial ASM cells, the growth medium was used instead of culture medium. The buffering capacity of the medium was adjusted by modification of the initial pH with Tris and MOPS base to achieve pH 7.4 at the target CO_2_ levels (pCO_2_ of 5%, 8%, and 20%, corresponding to 30–40, 50–60, and ~120 mmHg, respectively). Desired CO_2_ and pH levels were obtained by equilibration of the medium overnight in a humidified C-Chamber (BioSpherix), with CO_2_ controlled using a PRO CO_2_ carbon dioxide controller (BioSpherix). In this chamber, cells were exposed to the target pCO_2_ while maintaining 21% O_2_ balanced with N_2_. Before CO_2_ exposure, medium pH, pCO_2_, and pO_2_ were measured using a Stat Profile PRIME CCS analyzer (Nova Biomedical).

### Single-live-cell [Ca2+]i imaging

Mouse or human ASM cells stably expressing the genetically encoded calcium indicator GCaMP6s were generated using VSVG-pseudotyped lentivirus as previously described (57) with 293T packaging cells (Gene Hunter). ASM cells were sparsely plated on 40 mm glass coverslips and maintained in culture medium.

For imaging experiments, cells were incubated in an experimental medium prepared from a standard buffer (150 mM NaCl, 5 mM KCl, 1 mM MgCl_2_, 10 mM glucose, 25 mM sodium bicarbonate, with or without 2.5 mM CaCl_2_; pH 7.4) mixed with Ham’s F-12 medium, Tris base, and MOPS (3:1:0.25:0.25). The buffering capacity was adjusted by modification of the initial pH with Tris and MOPS to achieve extracellular pH of 7.4 or 7.2 at different CO_2_ concentrations. In some experiments, cells were infected with lentiviral scramble, *Stim1*, or *Orai1* shRNA.

Coverslips were mounted in an environmental control system chamber, and images were acquired using a Nikon TE2000U microscope with a digital camera controlled by NIS-Elements AR software (Nikon Instruments). Baseline recordings were obtained for 30 seconds under control conditions (30–40 mmHg CO_2_, pH 7.4) prior to switching to high-CO_2_-equilibrated medium for the indicated duration. No differences in baseline GCaMP6 fluorescence or resting [Ca^2+^]_i_ were observed under normocapnic conditions prior to hypercapnia. Images were captured at 15-second intervals. [Ca^2+^]_i_ was quantified using the Δ*F*/*F*_0_ method (58). For each experiment, single ASM cells were randomly selected for measurement.

GCaMP6 fluorescence is reported to be sensitive to significant changes in intracellular pH and can be quenched under acidic conditions (59, 60). To evaluate whether hypercapnia-associated acidification affected our measurements, we first assessed intracellular pH in cultured mouse ASM cells exposed to hypercapnia. High CO_2_ caused a mild and transient decrease in intracellular pH at 5 minutes coinciding with the onset of the [Ca^2+^]_i_ decrease observed under buffered hypercapnia (Supplemental Figure 2B). This acidification was significantly smaller in magnitude and duration than that induced by experimental respiratory acidosis. To directly test whether this minor acidification quenches GCaMP6 fluorescence, we expressed EGFP, the fluorescent core of GCaMP6, via lentiviral transduction in mouse ASM cells. EGFP fluorescence was unaffected by high-CO_2_ exposure (Supplemental Figure 2C), indicating that hypercapnia-induced acidification does not contribute to quenching of GCaMP6 fluorescence.

### Animals

Adult male and female C57BL/6J mice (9–11 weeks old; strain 000664) and male SMMHC-CreER^T2^ mice (9–11 weeks old; strain 019079) were obtained from The Jackson Laboratory. Mice harboring *loxP* sites flanking exon 2 of the *Stim1* gene (*Stim1^fl/fl^* mice) (provided by S. Feske, New York University, New York, New York, USA) were crossed with SMMHC-CreER^T2^ mice to generate *Myh11^CreERT2^ Stim1^fl/wt^* offspring. These heterozygous mice were then intercrossed to obtain homozygous *Myh11^CreERT2^ Stim1^fl/fl^* mice.

To induce smooth muscle–specific *Stim1* deletion, male *Myh11^CreERT2^ Stim1^fl/fl^* mice were given tamoxifen (100 μL of 10 mg/mL in sunflower oil) via intraperitoneal injection once daily for 5 consecutive days, as previously described (13, 41). Littermate *Myh11^CreERT2^ Stim1^fl/fl^* mice injected with vehicle (sunflower oil) served as controls. *Stim1*-deficient smooth muscle knockout (*Stim1*-smKO) mice were used for experiments 1 week after the final tamoxifen injection. Reduction of *Stim1* expression in tracheal tissue was confirmed by qPCR (Supplemental Figure 10).

All mice were maintained on a 14-hour light/10-hour dark cycle with free access to food and water and handled in accordance with NIH guidelines. Animals were euthanized using Euthasol (pentobarbital sodium–phenytoin sodium), and trachea or lung tissue was harvested for ASM cell isolation, qPCR, or preparation of PCLSs.

### Measurement of airway contraction in PCLSs

PCLSs were prepared as previously described (5). Briefly, following tracheotomy, excised mouse lungs were insufflated with 2.5% low-melting-point agarose in PBS and then placed in cold PBS to allow gelation. The right lobe was separated and sectioned into 100-μm-thick slices using a vibratome (VF-300, Precisionary Instruments). Lung slices were incubated at 37°C in culture medium, which was replaced hourly for 3 hours.

PCLS culture experiments were initiated by replacement of the existing medium with CO_2_-equilibrated medium, followed by incubation in a C-Chamber. In our previous studies (5), hypercapnic ASM cells exhibited maximal hyperreactivity and contractile responses after 2–7 days of exposure to high CO_2_ (~120 mmHg, extracellular pH 7.4). Based on these findings, PCLS experiments were conducted under 2-day high-CO_2_-exposure conditions (~120 mmHg, extracellular pH 7.4). After incubation, PCLS were transferred to the environmental control system chamber and exposed to 1 μM acetylcholine for 2 minutes. Changes in the airway lumen cross-sectional area were recorded using a Nikon TE2000U microscope (Nikon Instruments) equipped with a digital camera (Photometrics) controlled by NIS-Elements AR software (Nikon Instruments). Time-lapse images were captured at 10-second intervals and analyzed by pixel summation using ImageJ software (NIH). A decrease in lumenal cross-sectional area was interpreted as agonist-induced airway constriction.

### Human genetic analysis

#### Cohort study 1.

We conducted a case-control study using the NUgene biorepository at the Feinberg School of Medicine, Center for Genetic Medicine, at Northwestern University. The NUgene Project is a biorepository that integrates longitudinal medical information from participating patients at Northwestern Medicine–affiliated hospitals and outpatient clinics. It is also part of the Electronic Medical Records and Genomics (eMERGE) Network, which aims to leverage electronic medical records for genomic research. Participants’ DNA samples were linked to self-reported questionnaire data and continuously updated information from our electronic medical record (EMR) representing actual clinical care events. Using electronic phenotyping methods, we identified 1,029 subjects genotyped by whole-genome sequencing, comprising 201 COPD patients and 828 healthy controls, all of European ancestry. A subset of 118 COPD patients and 118 healthy controls were matched for age, sex, and smoking status as detailed in Supplemental Figure 11. COPD cases were defined based on the ICD-10 code J44.9, excluding individuals with a history of inpatient admissions or emergency room visits for COPD exacerbation. Control subjects had no recorded ICD-10 codes for COPD (J44) or asthma (J45) and had not visited a primary care provider in the past 2 years (Supplemental Figure 11).

In preliminary analyses, we observed differences in minor allele frequency (MAF) between our 2 control cohorts (828 controls: rs1561786 MAF 13.2%, rs3794050 MAF 12.4%, Supplemental Table 1; 118 controls: rs1561786 MAF 5.9%, rs3794050 MAF 5.1%, Table 1). Control subjects in the case-control study were significantly older and included a higher proportion of ever-smokers compared with those in the preliminary analysis cohort. To account for these differences, we performed conditional logistic regression analyses to calculate adjusted OR and corresponding *P* values for each variant in the matched case-control study (Table 1).

Participant characteristics, along with *STIM1* SNP genotype and MAF distributions, are summarized in Table 1 and Supplemental Table 1.

#### Cohort study 2.

We conducted a retrospective study of COPD patients evaluated at the Department of Internal Medicine at the University of Giessen Lung Center between 2014 and 2022. COPD was diagnosed based on the Global Initiative for Obstructive Lung Disease (GOLD) criteria during routine outpatient visits. Inclusion criteria required assessment of lung function by plethysmography, availability of blood gas measurements, and eligibility for genotyping. Blood gas measurements were obtained on the same day as pulmonary function testing, and contemporaneous PaCO_2_ values were used to classify patients as normocapnic or hypercapnic, reflecting CO_2_ status at the time of lung function assessment. Only patients classified as GOLD stage II to IV were included. A total of 74 genotyped smokers of European ancestry were identified (Supplemental Figure 11). Patient characteristics are summarized in Table 2.

### Statistics

Statistical methods are detailed in the figure legends and relevant sections of Methods. Sample sizes (*n*) used for statistical analyses are indicated in the corresponding figures. All analyses were performed using Prism (version 10.4.1, GraphPad Software) or R (version 4.3.2).

Comparisons between 2 groups were made using unpaired Student’s 2-tailed *t* tests assuming equal variances or Welch’s 2 tailed *t* tests when variances were unequal. For comparisons among multiple groups with equal variances, 1-way ANOVA followed by Tukey’s post hoc test was used. Variances were evaluated using the *F* test or Brown-Forsythe test. For qPCR data, 1-sample, 2-tailed *t* tests were performed against a hypothetical mean of 1 for the control group. The χ^2^ test was used for categorical data. Statistical outliers were identified and excluded using Grubbs’s test when appropriate.

For the case-control genetic analysis, individuals carrying at least one minor allele were compared with those homozygous for the major allele (dominant genetic model). Conditional logistic regression was performed using the clogit function in the *survival* package in R to estimate ORs and 95% CIs for the association between each genetic variant and COPD. Analyses were adjusted for BMI, and matching was accounted for by inclusion of strata(match_id) in the model. Airway resistance was analyzed using a 2-way ANOVA to assess the main effects of CO_2_ retention (normocapnia vs. hypercapnia) and genotype, as well as their interaction. Type III sums of squares were applied to account for unbalanced group sizes. Post hoc comparisons were conducted using uncorrected Fisher’s least significant difference test to evaluate changes in airway resistance between normocapnia and hypercapnia within each genotype.

Data are presented as mean ± standard error of the mean (SEM), and a 2-sided *P* value less than 0.05 was considered statistically significant.

### Study approval

All animal experiments were conducted in accordance with protocols approved by the Northwestern University Institutional Animal Care and Use Committee (IS00010662). Human cohort studies were approved by the Institutional Review Boards of Northwestern University (STU00010003, STU00221298) and the University of Giessen (266/11). All study participants were informed and provided written consent prior to study.

### Data availability

Data of the findings of this study are included in the main article and supplemental material (see Supporting Data Values file). Further information is available upon request.

## Author contributions

MS conceptualized the study. MS, FJMR, JAP, MJP, A Brauniger, LCW, and MP developed the methodology. MS, VK, JAP, MJP, MA, and NDM performed the investigations and acquired the data. MS and MP interpreted the data. MS wrote the original draft of the manuscript. VK, JAP, MJP, ET, MA, NDM, FJMR, EPC, WS, A Bharat, LCW, GRSB, EL, LAD, A Bharat, IV, MP, and JIS reviewed and edited the manuscript. A Bharat, IV, and JIS acquired funding for the study. A Bharat, GRSB, IV, and JIS provided resources.

## Conflict of interest

The authors have declared that no conflict of interest exists.

## Funding support

This work was supported in whole or in part by the NIH and is subject to the NIH Public Access Policy. Through acceptance of this federal funding, the NIH has been granted the right to make the work publicly available in PubMed Central.

- NIH R01HL147070 to JIS, R01HL173987 to JIS and LAD, R01HL131745 to A Bharat, R01HL145478 to A Bharat, R01HL147290 to A Bharat, R01HL147575 to A Bharat, R01HL173940 to AB, P01HL169188 to A Bharat.
- Federal Ministry of Education and Research (German Center for Lung Research [DZL]/ALI 1.5, 3.3 and 3.4) to IV.
- von Behring Röntgen Foundation (Project 66-LV07) to IV.
- Science Foundation Ireland (SFI 15/CDA/3490) to EPC.

## Supplementary Material

Supplemental data

Unedited blot and gel images

Supplemental video 1

Supporting data values

## Figures and Tables

**Table 1:**
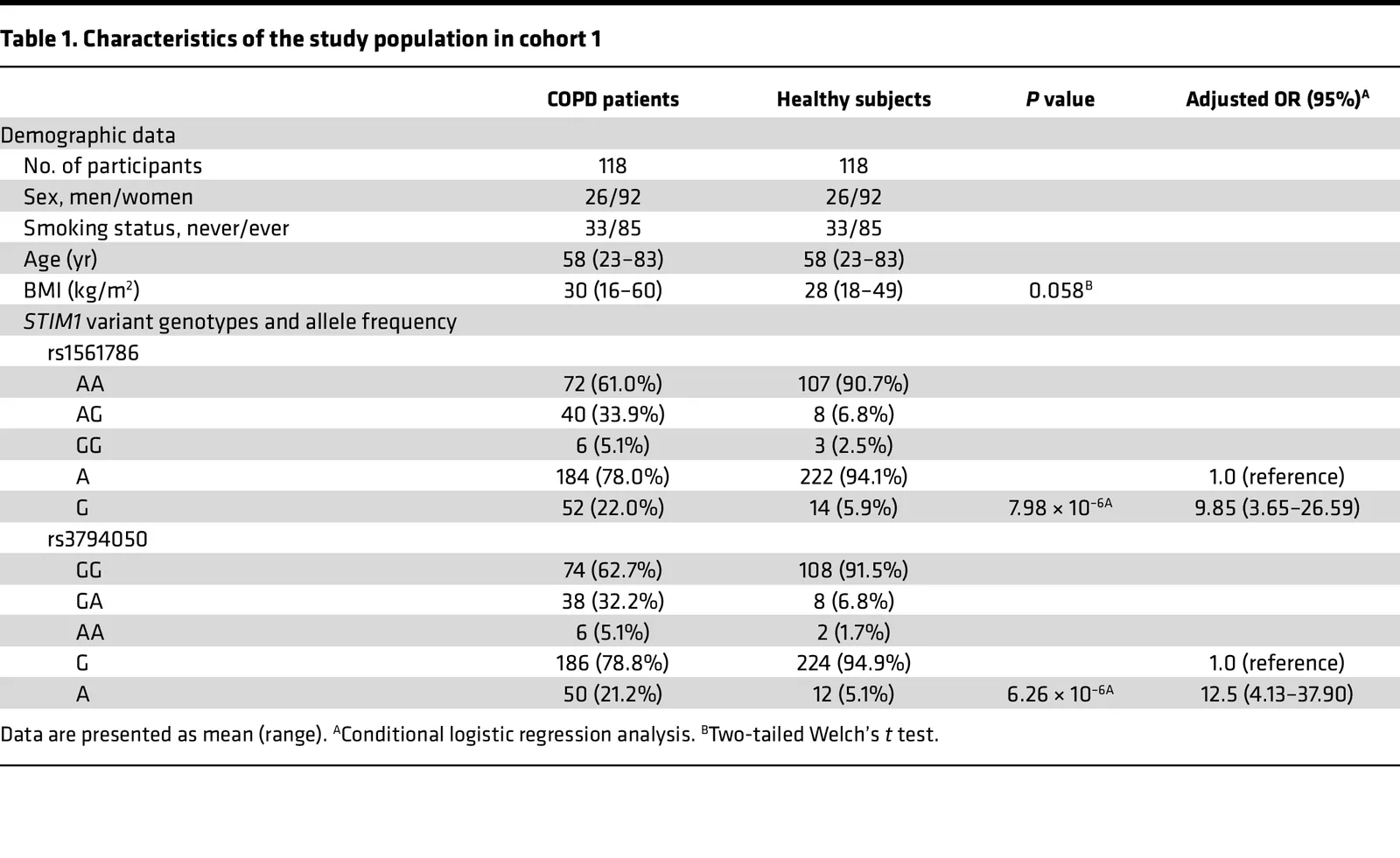
Characteristics of the study population in cohort 1

**Table 2:**
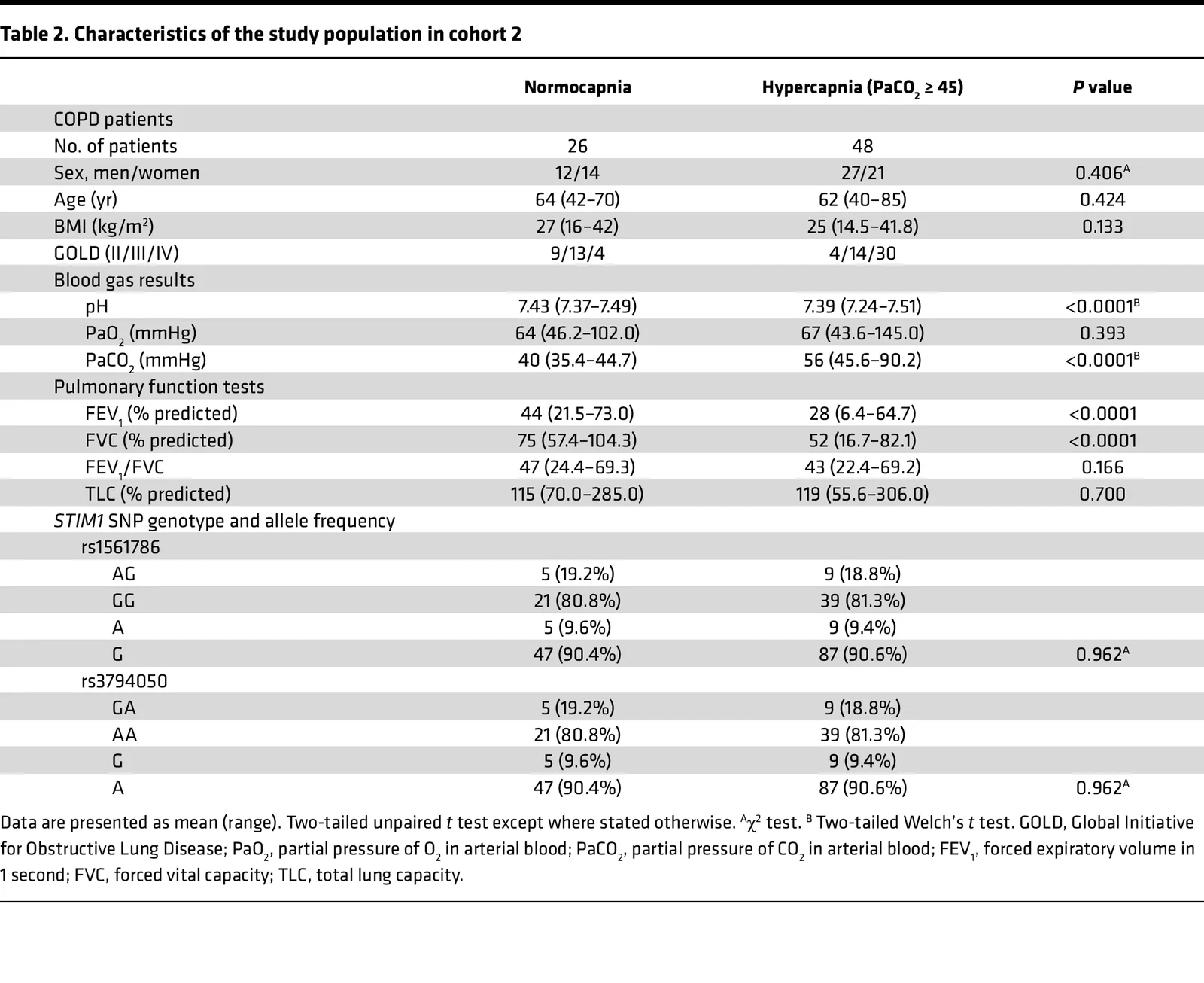
Characteristics of the study population in cohort 2

## Version 1 07/16/2026

In-Press Preview

## Version 2 09/08/2026

Electronic publication
