# Supplementary material for: Hypercapnia enhances airway smooth muscle contractility via STIM1-dependent Ca2+ signaling
Source: JCI Insight. 2026 Jul 16;11(17):e202475. doi: 10.1172/jci.insight.202475 (PMC13564089; doi:10.1172/jci.insight.202475)
Supplement: Supplementary file 1 [file jciinsight-11-202475-s118.pdf]

Supplemental data for:

Hypercapnia enhances airway smooth muscle contractility via STIM1-dependent Ca^{2+} signaling

Masahiko Shigemura[1,2*], Vitalii Kryvenko[3], Jennifer A. Pacheco[4], Megan J. Puckelwartz[4,5], Milos Aleksic[6], Natalia D. Magnani[7], Emma Thompson[8], Francisco Javier Martin-Romero[9], Eoin P. Cummins[10], Werner Seeger[3], Andreas Bräuninger[11], Lynn C. Welch[12], G.R. Scott Budinger[12], Emilia Lecuona[1], Laura A. Dada[12], Ankit Bharat[1], István Vadász[3], Murali Prakriya[5], and Jacob I. Sznajder[12]

Affiliations:

[1] Division of Thoracic Surgery, Feinberg School of Medicine, Northwestern University, Chicago, IL, USA

[2] Laboratory of Homeostatic Physiology, Department of Physiology and Cell Biology, Graduate School of Medicine, Kobe University, Kobe, Japan

[3] Department of Internal Medicine, Justus Liebig University, Universities of Giessen and Marburg Lung Center, German Center for Lung Research, The Cardio-Pulmonary Institute, and Institute for Lung Health, Giessen, Germany

[4] Center for Genetic Medicine, Feinberg School of Medicine, Northwestern University, Chicago, IL, USA

[5] Department of Pharmacology, Feinberg School of Medicine, Northwestern University, Chicago, IL, USA

[6] Department of Neuroscience, Feinberg School of Medicine, Northwestern University, Chicago, IL, USA

[7] Faculty of Pharmacy and Biochemistry, University of Buenos Aires, Buenos Aires, Argentina

[8] Department of Human Genetics, University of Chicago, Chicago, IL, USA

[9] Department of Biochemistry and Molecular Biology, Universidad de Extremadura, Badajoz, Spain

[10] University College Dublin School of Medicine; UCD Conway Institute of Biomolecular and Biomedical Research, Dublin, Ireland

[11] Institute of Pathology, Justus Liebig University, Giessen, Germany

[12] Division of Pulmonary and Critical Care Medicine, Feinberg School of Medicine, Northwestern University, Chicago, IL, USA

*** Address Correspondence to:** Masahiko Shigemura. Division of Thoracic Surgery, Feinberg School of Medicine, Northwestern University, Chicago, IL, USA. Phone: 1.312.503.5033; Email: masahiko.shigemura@northwestern.edu

This file contains:

Methods
Supplemental references
Supplemental Figure 1-11
Supplemental Table 1-2

Methods

Infection of lentiviral vector expressing *Stim1* or *Orai1* shRNA

Cell culture and generation of mouse airway smooth muscle (ASM) cells with stable knockdown of *Stim1*, *Orai1* or a scrambled shRNA control were performed using the Sigma Mission lentiviral packaging mix (Sigma-Aldrich) with the following catalog numbers: STIM1 (TRCN0000193400), ORAI1 (TRCN0000125405), non-target control (SHC312). Transformed cells were cultured with 2 µg/ml puromycin for 2 days for selection and reduction in the expression of the target gene was assessed by Western blot (Supplemental Figure 8).

Infection of lentiviral vector expressing enhanced GFP (eGFP)

To generate cells lines of mouse ASM cells stably expressing eGFP, the VSVG pseudotyped lentivirus expressing pGIPZ (Open Biosystems) was generated as described elsewhere (1) (DNA/RNA Delivery Core, SDRC, Northwestern University) using 293T packaging cells (Gene Hunter).

Measurement of intracellular pH

Intracellular pH level in cultured mouse ASM cells exposed to different high CO_2 conditions with the extracellular pH of 7.4 or 7.2 was determined using pHrodo™ Green AM Intracellular pH Indicator (Thermo Fisher Scientific) according to the manufacturer's instructions. Fluorescence intensity was measured at 509 nm excitation and 533 nm emission wavelengths using a microplate reader (SpectraMAX Microplate Reader, Molecular Devices).

Transfection of mCherry-tagged STIM1 plasmids

Cultured mouse ASM cells expressing lentiviral *Stim1* shRNA or human ASM cells were transiently transfected either with 1 µg of mCherry-tagged full-length STIM1 or mutant STIM1$^{S575A/S608A/S621A}$ plasmid (2, 3) by using Lipofectamine 2000 following the manufacturer's recommended protocol. Experiments were performed 24 h after transfection.

Single cell-live imaging of mCherry-tagged STIM1

Cultured mouse ASM cells transfected with mCherry-tagged STIM1 were sparsely plated and cultured onto 40 mm glass coverslips and incubated in culture medium: DMEM supplemented with 10% FBS, penicillin (100 U/ml), and streptomycin (100 µg/ml). Experimental conditions such as CO_2-equilibrated standard buffer medium and microscopic system are described in Single cell-live $[Ca^{2+}]_i$ imaging in the Method section. An initial recording (time, 0 min) was made to obtain the mCherry-tagged STIM1 signal in cells exposed to control (30-40 mmHg CO_2, pH 7.4) 30 s before high CO_2 exposure. Experiment was started by switching to high CO_2-equilibrated standard buffer medium and incubating in the environmental control system chamber for the specified time. The images were analyzed with ImageJ (NIH) software (4, 5). They were threshold to remove background fluorescence and used to create a mask image (5). Using the mask images, number of

mCherry-tagged STIM1 clusters was scored with automatic “Analyze Particles” algorithm of ImageJ software and using cluster size of 3–100 pixels and circularity 0.1–1.0. The data were expressed as the number of clusters per area (μm^2) of the cell (4).

Transfection of siRNAs

Mouse ASM cells were plated at a density of 1.1×10^5 cells per well in six-well plates with DMEM containing 10% FBS without any antibiotics (6). The next day, the cultured cells were transfected with 25 nM siRNAs (siGENOME SMARTpool-Fos, Dharmacon) using the DharmaFECT 1 Transfection Reagent (Dharmacon), according to the manufacturer’s instructions. A siGENOME nontargeting siRNA pool 1 (Dharmacon) was used as a negative control. At 24 hours after transfection, the cells were cultured in the CO_2 medium for the desired time and were harvested. Reduction in the expression of the target molecule was assessed in Supplemental Figure 9.

Cell lysate preparation and Western blot analysis

Cells were lysed in cell lysis buffer (Cell Signaling Technology), and protein concentrations were determined using the Bradford assay (Bio-Rad). Equal amounts of protein were separated on 7.5%, 12.5%, or 4–20% SDS–polyacrylamide gels and transferred onto nitrocellulose membranes (Bio-Rad) using a Trans-Blot Turbo Transfer System (Bio-Rad). Membranes were incubated overnight at 4°C with primary antibodies against STIM1 (Cell Signaling Technology, #5668), STIM2 (Cell signaling Technology, #4917) Orai1 (ProSci, #4281), β-Tubulin (Cell Signaling Technology, #2146), phospho-ERK (Cell Signaling Technology, #9101), total ERK (Cell Signaling Technology, #9107), RhoA (Cell Signaling Technology, #2117), c-Fos (Cell Signaling Technology, #4384) and α-SMA (R&D systems, MAB1420). β-Tubulin and total ERK were used as loading controls. HRP-conjugated secondary antibodies (Cell Signaling Technology, #7074, #7076) were applied, and immunoreactive bands were detected using the SuperSignal™ West Femto Maximum Sensitivity Substrate (Thermo Fisher Scientific) according to the manufacturer’s instructions. Chemiluminescent signals were imaged and quantified using a LI-COR Odyssey Fc Imaging System and Image Studio software (version 5.2; LI-COR Biosciences) or ImageJ (NIH).

Nuclear fractionation

Cells were harvested, and nuclear/cytoplasmic fractionation was performed using a commercially available kit (BioVision) according to the manufacturer’s protocol. Nuclear proteins were separated by SDS–PAGE, transferred to nitrocellulose membranes, and probed with antibodies against phospho-c-Fos (Ser374) (Invitrogen, #PA5-105995), total c-Fos (Cell Signaling Technology, #4384), cleaved caspase-7 (Cell Signaling Technology, #8438), and histone H3 (Cell Signaling Technology, #4499). Histone H3 was used as a nuclear loading control.

Co-Immunoprecipitation (Co-IP)

Cells were lysed in cell lysis buffer (Cell Signaling Technology), and protein concentrations were measured using the Bradford assay (Bio-Rad). Equal amounts of lysate (250 μg for STIM1 IP/Orai1 blotting; 1–1.5 mg for other IP/blotting experiments) were incubated overnight at 4°C with the indicated antibodies: phospho-MAPK/CDK Substrates (PXS*P or S*PXR/K) (Cell Signaling Technology, #2325), STIM1 (Cell Signaling Technology, #5668) or mCherry (Thermo Scientific, #PA5-34974). Protein A magnetic beads (Cell Signaling Technology) were then added and incubated for 20 minutes at room temperature. Beads were washed five times with lysis buffer, resuspended in 3× Laemmli sample buffer, and analyzed by SDS–PAGE. Immunoblotting was performed using antibodies against IP3 receptor 1 (Invitrogen, #PA1-901), phospho-MAPK/CDK Substrates (PXS*P or S*PXR/K) (Cell Signaling Technology, #2325), mCherry (Thermo Scientific, #PA5-34974), Orai1 (ProSci, #4281) and STIM1 (Cell Signaling Technology, #5668), following by HRP-conjugated secondary antibody (Cell Signaling Technology, #5127).

Chromatin immunoprecipitation (ChIP)

Protein–DNA interactions were analyzed using the CUT&RUN Assay Kit (Cell Signaling Technology) according to the manufacturer's instructions. Briefly, 1.5–2.5 × 10^5 cells were used per reaction. Cells were washed, bound to concanavalin A–coated magnetic beads, and permeabilized with Dig Wash Buffer containing 0.05% digitonin. The cell–bead complexes were incubated overnight at 4°C with antibodies against c-Fos (Cell Signaling Technology, #2250) or control rabbit IgG (Cell Signaling Technology, #66362) at a 1:50 dilution. After washing with Dig Wash Buffer, Protein A–MNase was added and incubated for 1 hour at 4°C. Digestion was initiated by adding 2 mM $CaCl_2$ and incubating on ice for 30 minutes. The reaction was terminated by adding an equal volume of 2× Stop Buffer (20 mM EDTA, 0.05% digitonin, 5 mg/mL RNase A, and 2 pg/mL heterologous spike-in DNA). CUT&RUN fragments were released by incubation at 37°C for 30 minutes and centrifuged at 16000 × g for 5 min. DNA was extracted from the supernatant using DNA Purification Buffers and Spin Columns (Cell Signaling Technology).

Recovered DNA were quantified by qPCR as described previously (6). Primers were designed based on computational prediction of c-Fos–binding sites using JASPAR (http://jaspar.genereg.net): forward, 5'-AGGGCAGTAGCTGTCAC-3'; reverse, 5'-CCAGCCTCCAGCTCTAA-3'. Data are presented as fold enrichment relative to control IgG (7).

Measurement of $[Ca^{2+}]_i$ in ASM cells exposed to prolonged hypercapnia

Cultured cells grown on 40-mm coverslips were exposed to control or high CO_2 medium for up to 18 hours. Cells were then loaded with 2 μM Fura-2–acetoxymethyl ester (Fura-2 AM; Life Technologies) in the presence of 0.05% Pluronic F-127 (Life Technologies) and 2.5 mM probenecid (Sigma) for 30 min at room temperature in standard buffer solution (150 mM NaCl, 5 mM KCl, 1 mM $MgCl_2$, 10 mM glucose, 25 mM $NaHCO_3$, and 2.5 mM $CaCl_2$; pH 7.4) in the dark.

After loading, cells were washed with PBS and incubated for an additional 30 min at room temperature to allow complete de-esterification of the dye.

Fura-2 fluorescence was excited at 340 and 380 nm, and emission was collected at 510 nm using a Nikon TE2000U inverted microscope (Nikon Instruments) equipped with an environmental control chamber (FCS2 system; Bioptechs) and a Plan Super Fluor 40× oil objective. Images were captured with a Cascade EMCCD camera (model TC285; Photometrics) and analyzed using MetaFluor software (Molecular Devices). Changes in $[Ca^{2+}]_i$ were calculated from the F_{340}/F_{380} fluorescence ratio.

Measurement of calpain activity

Calpains activity in total cell lysates was determined using a Calpain Activity Assay it (Biovision) according to the manufacturer's instructions. Briefly, mouse ASM cells were collected and lysed in extraction buffer. Equal amounts of protein were added to the calpain substrate Ac-LLY-AFC. Fluorescence intensity indicating calpains activity was measured at 400 nm excitation and 505 nm emission wavelengths using a microplate reader (SpectraMAX M2e Microplate Reader, Molecular Devices).

qPCR

Total RNA was isolated from cultured cells homogenized in 700 µL of lysis/binding buffer using the miRNeasy Mini Kit (Qiagen) according to the manufacturer's instructions. cDNA was synthesized from 1 µg of total RNA using the qScript cDNA Synthesis Kit (Quanta Biosciences). qPCR was performed using SYBR Green chemistry (Bio-Rad) with gene-specific primers (Integrated DNA Technologies).

The following primer pairs were used:

Rpl19, forward 5'-GAAGGTCAAAGGGAATGTGTTCAA-3', reverse 5'-TTTCGTGCTTCCTTGGTCTTAGA-3'; *Stim1*, forward 5'-CTGAAGATGACAGATCGGAGC-3', reverse 5'-ACCCACACCAATAACGATAGAC-3'.

Relative transcript expression was calculated using the ΔΔCt method with *Rpl19* as the internal reference gene for normalization.

Measurement of cell contraction

Cell contraction was assessed according to the technique described elsewhere (6, 8). Cultured ASM cells were sparsely plated onto 40 mm glass coverslips and exposed to different CO_2 conditions for 2 days. The coverslip was mounted in an environmental control system chamber and images were taken using a Nikon TE2000U microscope equipped with a digital camera driven by NIS Element AR software (Nikon Instruments). An initial recording (time, 0 min) was made to obtain the size of quiescent cells. Experiments were started by replacing the culture medium with the CO_2-equilibrated medium and incubating in the environmental control system chamber for the

specified time. For the agonist-induced contraction experiment, acetylcholine (ACh) was added to the chamber, and images were recorded at 10 second intervals for 5 min. Cell contraction was calculated as the ratio of the change in surface area to the initial value using ImageJ (NIH).

In our preliminary experiments using mouse ASM cells under normocapnic conditions (6), ACh-induced cell contraction was induced in a dose-dependent manner. Based on this result, 1 μM of ACh, which is the lowest concentration to induce cell contraction, was selected for subsequent experiments.

Cell proliferation

Mouse ASM cells expressing non-targeting or *Stim1* shRNA were plated at a density of 0.5×10^5 cells per 60-mm dish in complete medium. After 24 hours, cells were exposed to control CO_2 conditions (30–40 mmHg, pH 7.4) or buffered hypercapnia (~120 mmHg CO_2, pH 7.4) for the indicated durations. Cell proliferation was quantified as the percentage increase in cell number relative to the number of cells initially plated.

eQTL Analysis

We assessed the association between *STIM1* SNP genotypes and gene expression using the GTEx Portal (https://gtexportal.org/), a publicly available resource of tissue-specific gene expression and genetic variation data. Expression quantitative trait loci (eQTL) analysis was performed specifically in lung tissue to evaluate whether the selected *STIM1* SNPs (rs1561876, rs3750994, rs3750996, rs3794050 and rs7934581) were associated with differences in *STIM1* expression levels. Among these, rs3750994 and rs3750996 were not identified as eQTL in lung tissue. For rs7934581, the T allele is insufficiently represented in the GTEx datasets adequately to make any statements regarding its effect on expression. Genotype-specific expression patterns were visualized using the GTEx eQTL calculator (Figure 7C).

Single-cell RNA-seq analysis of *STIM1* expression

STIM1 expression was analyzed using the publicly available COPD Cell Atlas single-cell RNA-sequencing dataset (GSE136831) (9). Raw unique molecular identifier count matrices, cell barcodes, gene identifiers, and published cell-type annotations were downloaded from GEO and assembled into a single expression object (312,928 cells × 45,947 genes) using Scanpy (v1.11.5) and AnnData in Python (v3.11.15). Counts were normalized to 10,000 per cell and log-transformed (log1p). Cell-type identities and lineage categories were assigned according to the published dataset annotations.

For per-subject smooth-muscle analysis (Figure 7A), smooth-muscle cells (SMC) were identified using the published "SMC" cell-type label (706 cells, all within the stromal compartment). Identity was confirmed by enrichment of canonical smooth-muscle markers (*ACTA2*, *MYH11*, *TAGLN*, and *DES*) relative to all other cell populations. For each donor, *STIM1* expression was summarized as

the mean log-normalized value across that donor's SMC, and per-donor means were compared between control and COPD donors.

For the cell-type survey (Supplemental Figure 6), *STIM1* expression was summarized for each cell type within each disease group as the mean log-normalized expression across all cells of that type and the percentage of cells with detectable expression (>0 counts). Cell types were grouped into epithelial, endothelial, stromal, and immune (myeloid and lymphoid) lineages according to the published dataset annotations, and multiplet-annotated cells were excluded.

References

1. Zufferey R, Dull T, Mandel RJ, Bukovsky A, Quiroz D, Naldini L, et al. Self-inactivating lentivirus vector for safe and efficient in vivo gene delivery. *Journal of virology.* 1998;72(12):9873-80.
2. Pozo-Guisado E, Campbell DG, Deak M, Alvarez-Barrientos A, Morrice NA, Alvarez IS, et al. Phosphorylation of STIM1 at ERK1/2 target sites modulates store-operated calcium entry. *J Cell Sci.* 2010;123(Pt 18):3084-93.
3. Pozo-Guisado E, Casas-Rua V, Tomas-Martin P, Lopez-Guerrero AM, Alvarez-Barrientos A, and Martin-Romero FJ. Phosphorylation of STIM1 at ERK1/2 target sites regulates interaction with the microtubule plus-end binding protein EB1. *J Cell Sci.* 2013;126(Pt 14):3170-80.
4. Yazbeck P, Tauseef M, Kruse K, Amin MR, Sheikh R, Feske S, et al. STIM1 Phosphorylation at Y361 Recruits Orai1 to STIM1 Puncta and Induces Ca(2+) Entry. *Sci Rep.* 2017;7:42758.
5. Dagda RK, Zhu J, Kulich SM, and Chu CT. Mitochondrially localized ERK2 regulates mitophagy and autophagic cell stress: implications for Parkinson's disease. *Autophagy.* 2008;4(6):770-82.
6. Shigemura M, Lecuona E, Angulo M, Homma T, Rodriguez DA, Gonzalez-Gonzalez FJ, et al. Hypercapnia increases airway smooth muscle contractility via caspase-7-mediated miR-133a-RhoA signaling. *Sci Transl Med.* 2018;10(457).
7. Decourcelle A, Very N, Djouina M, Loison I, Thevenet J, Body-Malapel M, et al. O-GlcNAcylation Links Nutrition to the Epigenetic Downregulation of UNC5A during Colon Carcinogenesis. *Cancers (Basel).* 2020;12(11).
8. Jiang H, Xie Y, Abel PW, Toews ML, Townley RG, Casale TB, et al. Targeting phosphoinositide 3-kinase γ in airway smooth muscle cells to suppress interleukin-13-induced mouse airway hyperresponsiveness. *J Pharmacol Exp Ther.* 2012;342(2):305-11.

9. Sauler M, McDonough JE, Adams TS, Kothapalli N, Barnthaler T, Werder RB, et al. Characterization of the COPD alveolar niche using single-cell RNA sequencing. *Nat Commun.* 2022;13(1):494.

Supplemental figures and tables

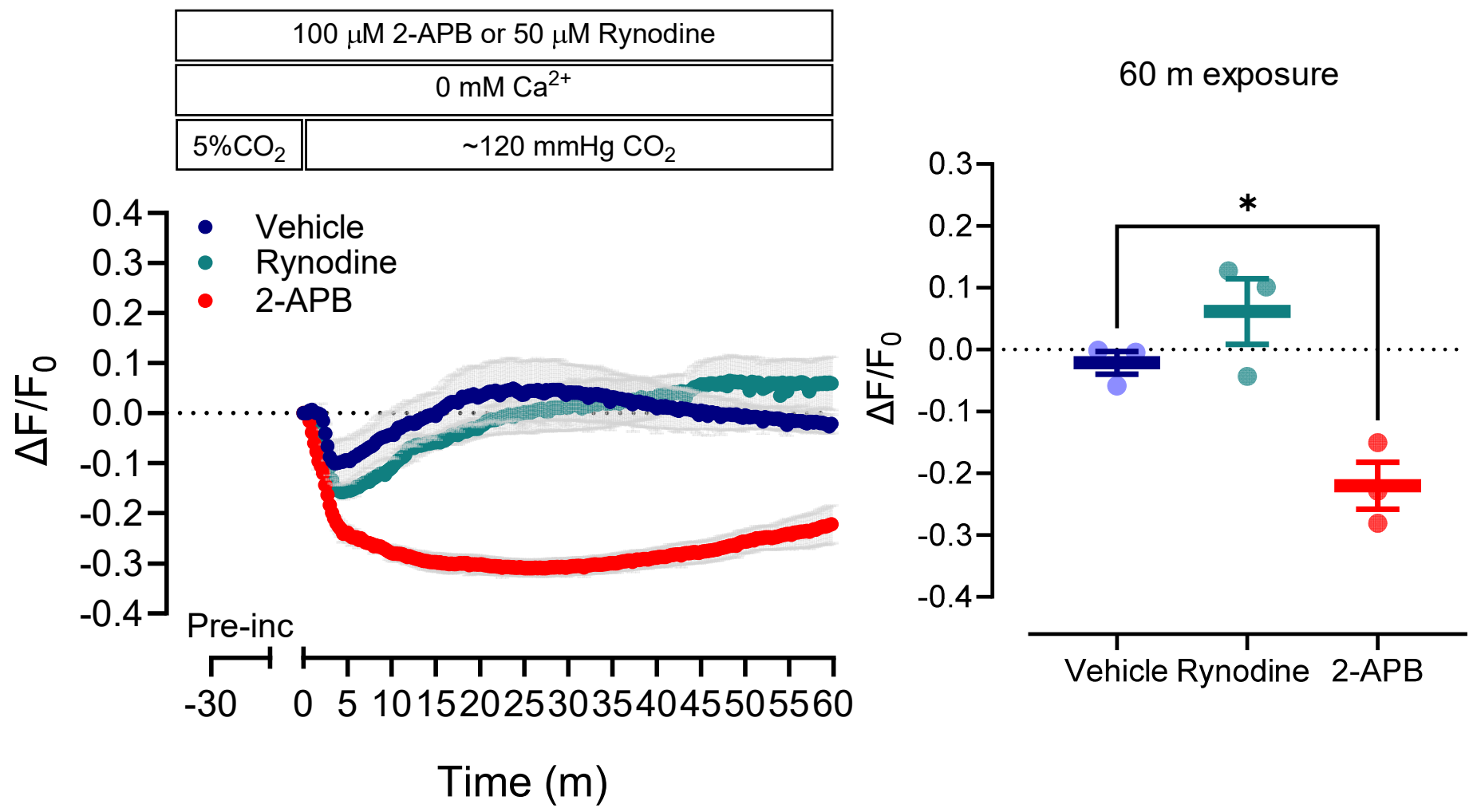

Supplemental Figure 1. High CO_2 induces sarcoplasmic reticulum Ca^{2+} depletion via inositol trisphosphate receptor.

Intracellular Ca^{2+} dynamics were assessed in cultured mouse ASM cells expressing GCaMP6 (n=3 biological replicates). $[Ca^{2+}]_i$ traces (left) and quantification of $\Delta F/F_0$ at 60 min (right) are shown under Ca^{2+}-free conditions. Cells were preincubated for 30 min with DMSO (vehicle), 100 µM 2-APB or 50 µM Ryanodine in 5% CO_2 (30-40 mmHg, pH 7.4)-equilibrated medium, followed by exposure to high CO_2 (~120 mmHg, pH 7.4) in the continued presence of the inhibitor for 60 min.

Data are presented as mean ± SEM. Statistical analysis was performed by one-way ANOVA with Tukey's post hoc test. $^*p < 0.05$.

Pre-inc, preincubation.

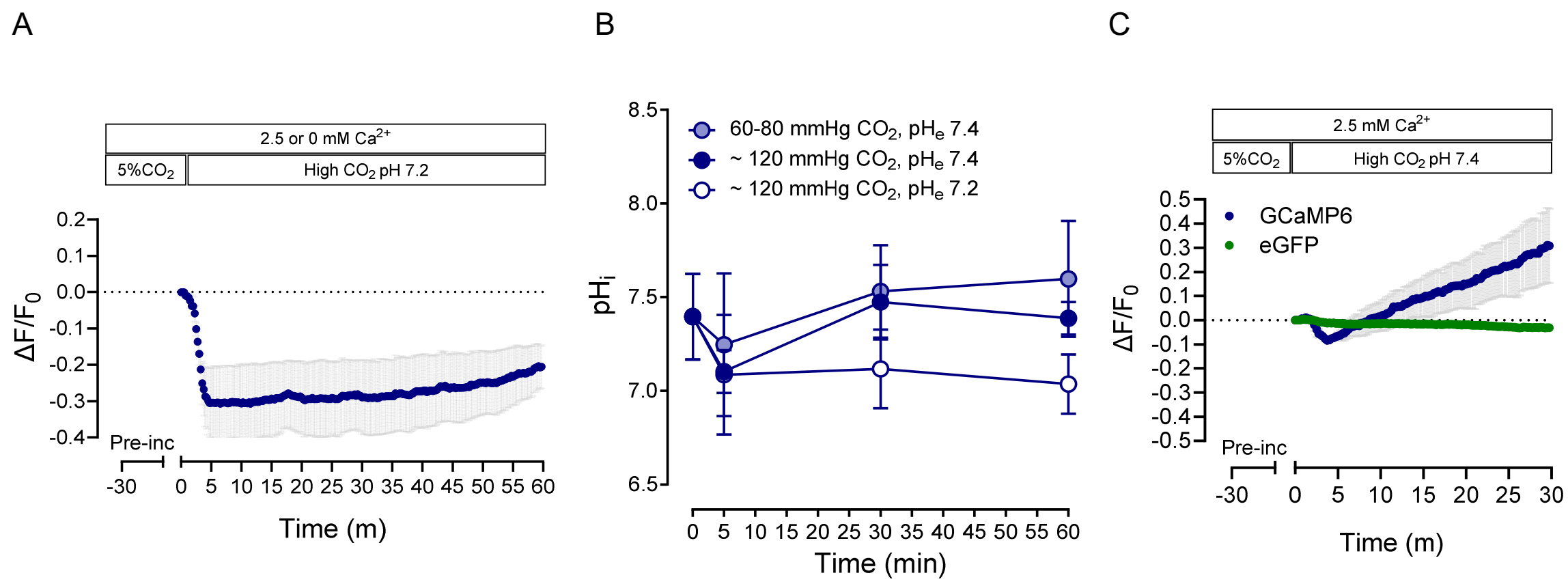

Supplemental Figure 2. Effect of intracellular acidosis on $[Ca^{2+}]_i$ dynamics in ASM cells measured using GCaMP6.

(**A**) $[Ca^{2+}]_i$ traces in cultured mouse ASM cells exposed to conditions mimicking respiratory acidosis (n=3 biological replicates). Cells were preincubated for 30 min in 5% CO_2 (30-40 mmHg, pH 7.4) medium, subsequently exposed for 60 min to high CO_2 (~120 mmHg, pH 7.2) in the presence of 2.5 mM Ca^{2+}. (**B**) Intracellular pH (pH_i) levels in cultured mouse ASM cells exposed to different high CO_2 conditions with extracellular pH (pH_e) 7.4 or 7.2 (n=3 biological replicates). (**C**) eGFP fluorescence in cultured mouse ASM cells exposed to high CO_2 (n=3 biological replicates). Cells were preincubated for 30 min in 5% CO_2 medium and then exposed for 30 min to high CO_2 (~120 mmHg, pH 7.4) in the presence of 2.5 mM Ca^{2+}. GCaMP6 data shown in this panel are derived from Figure 1A.

Data are presented as means ± SEM.

Pre-inc, preincubation.

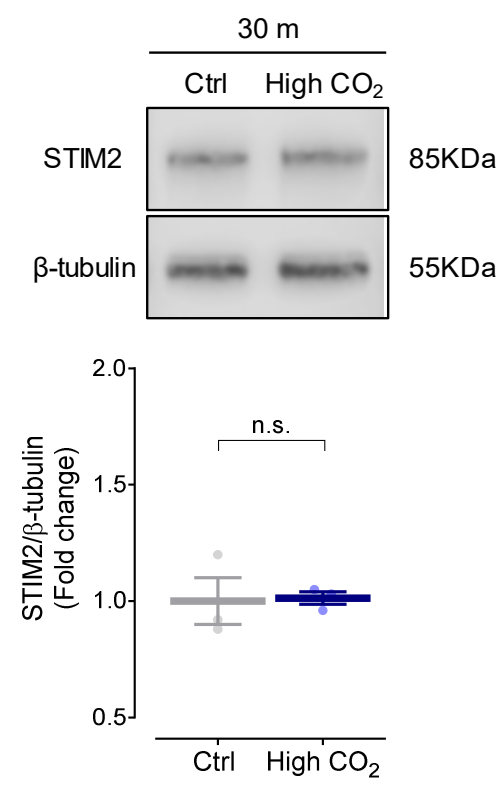

Supplemental Figure 3. STIM2 expression in mouse ASM cells exposed to hypercapnia.

Cultured mouse ASM cells were incubated in control CO_2 conditions (Ctrl; 30-40 mmHg, pH 7.4) or exposed to high CO_2 (~120 mmHg, pH 7.4) for 30 min. Representative Western blot (top) and quantification (bottom) of STIM2 (n=3 independent biological replicates).

Data are presented as mean ± SEM. Statistical analysis was performed by unpaired *t*-test. n.s., not significant.

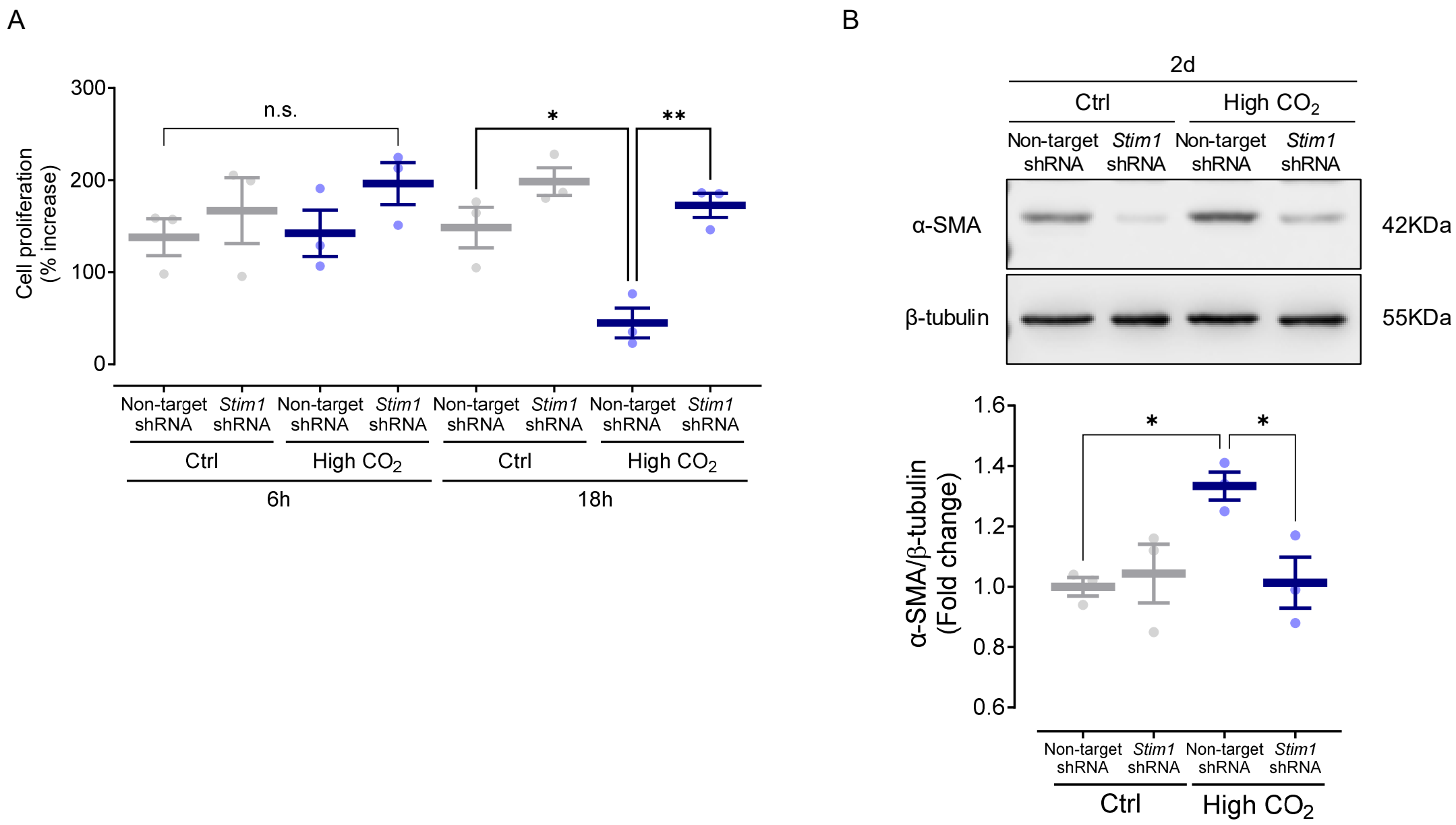

Supplemental Figure 4. Hypercapnia increases α-smooth muscle actin (α-SMA) expression and impairs ASM proliferation in a STIM1-dependent manner.

Cultured mouse ASM cells expressing non-targeting or *Stim1* shRNA were incubated in control CO_2 conditions (Ctrl; 30~40 mmHg, pH 7.4) or exposed to high CO_2 (~120 mmHg, pH 7.4) for the indicated durations. (**A**) Cell proliferation, quantified as the percentage increase relative to the number of cells initially plated. (**B**) Representative Western blots (top) and quantification (bottom) of α-SMA expression.

Data are presented as mean ± SEM. Statistical analysis was performed by one-way ANOVA with Tukey's post hoc test. *$p < 0.05$, **$p < 0.01$, n.s., not significant.

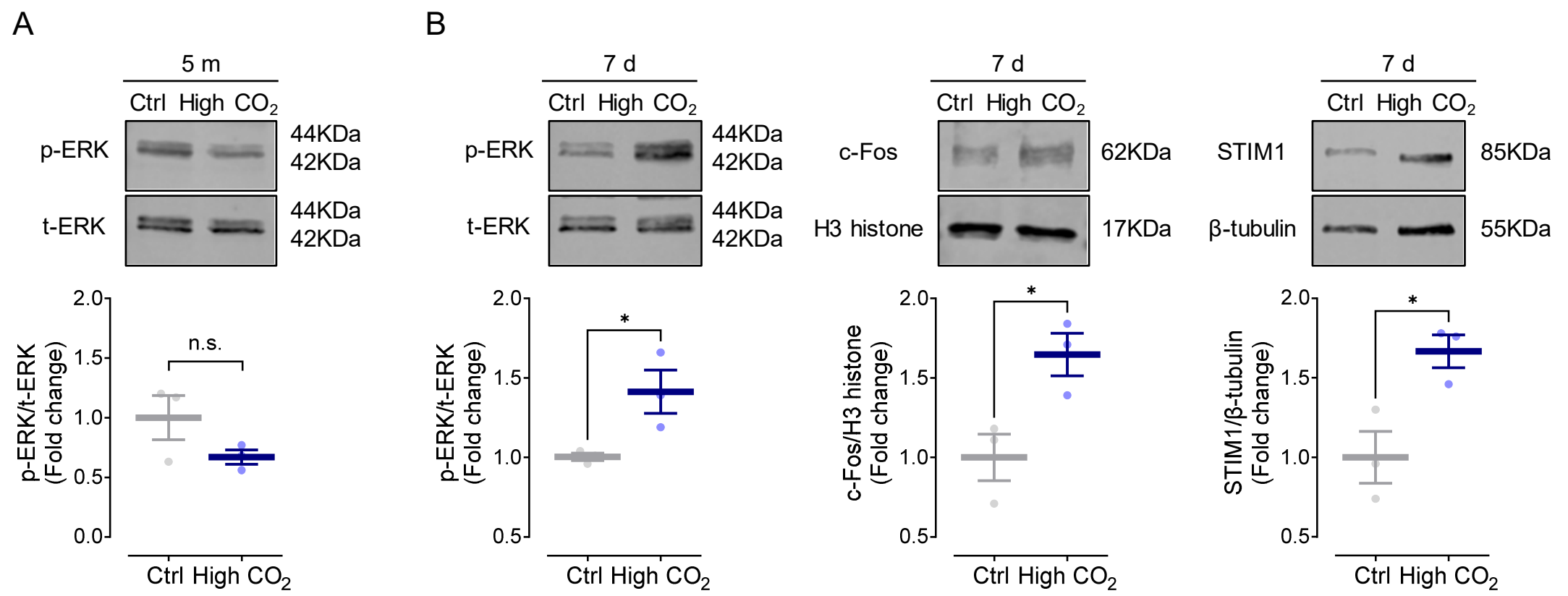

Supplemental Figure 5. Clinically relevant hypercapnia activates the ERK/c-Fos/STIM1 signaling axis in ASM cells.

Cultured ASM cells were incubated in control CO_2 conditions (Ctrl; 30-40 mmHg, pH 7.4) or exposed to high CO_2 (50-60 mmHg, pH 7.4) for the indicated time. (**A**) Representative Western blot (top) and quantification (bottom) of phosphorylated ERK (p-ERK) in human ASM cells (n=3 independent experimental replicates). (**B**) Representative Western blot (top) and quantification (bottom) of p-ERK (left), nuclear c-Fos (middle) and STIM1 (right) in mouse ASM cells (n=3 independent biological replicates).

Data are presented as mean ± SEM. Statistical analysis was performed by unpaired *t*-test. *$p < 0.05$, n.s., not significant.

t-ERK, total ERK.

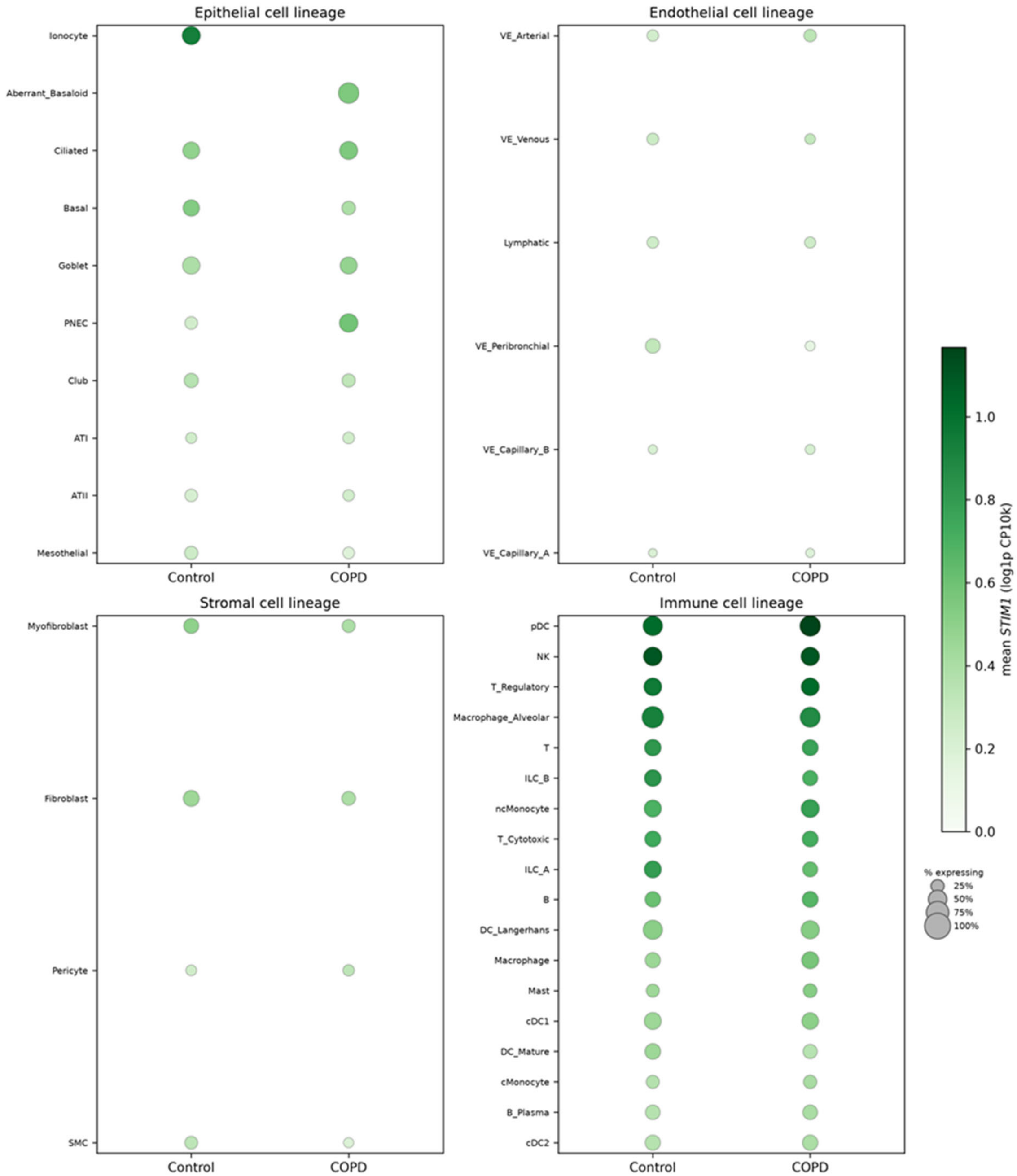

Supplemental Figure 6. Single-cell RNA-seq analysis of *STIM1* expression in lung cells from explanted COPD lungs and donor control lungs.

STIM1 expression was analyzed by previously published single-cell RNA-sequencing datasets from explanted COPD lungs and controls (GSE136831) (9). For each cell type, dot color indicates the mean log-normalized *STIM1* expression and dot size indicates the percentage of cells with detectable *STIM1*, shown separately for control and COPD samples. Color scaling was kept consistent across all panels.

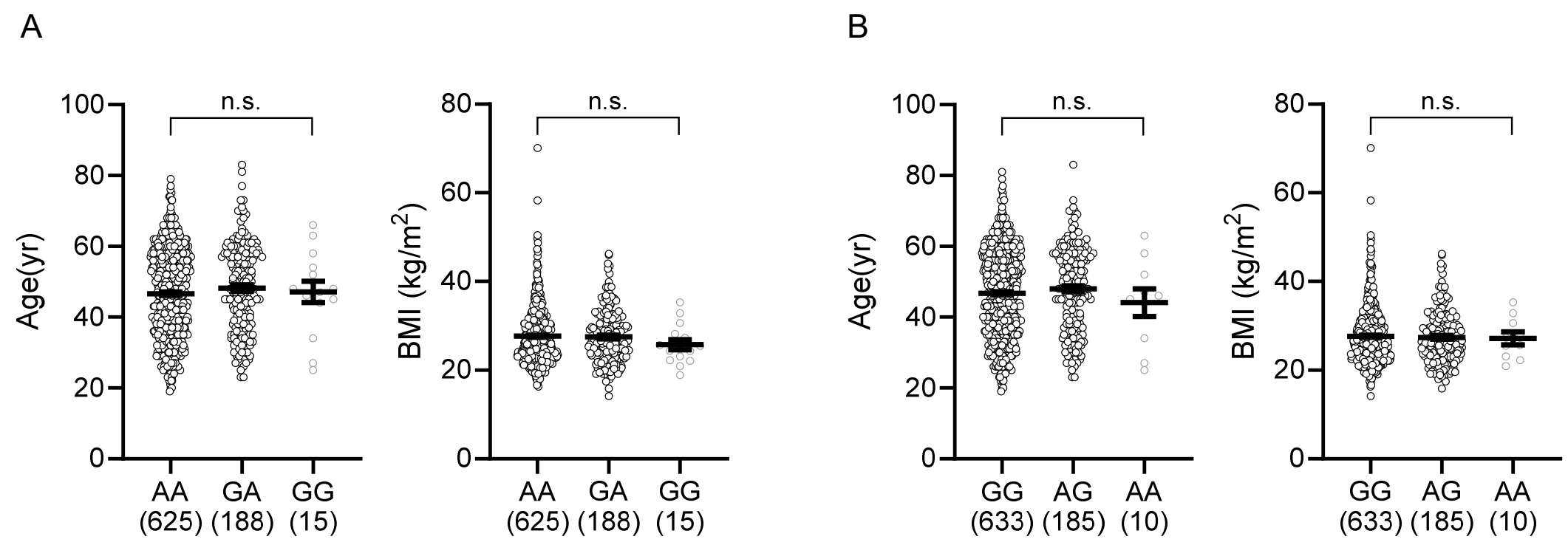

Supplemental Figure 7. Effects of age and BMI on the prevalence of rs1561876 and rs3794050 in 828 individuals of European ancestry.

(**A**) Prevalence of rs1561876. (**B**) Prevalence of rs3794050.

Data are presented as means ± SEM. Statistical analysis was performed by one-way ANOVA with Tukey's post hoc test. Sample sizes are indicated in the figure. n.s., not significant.

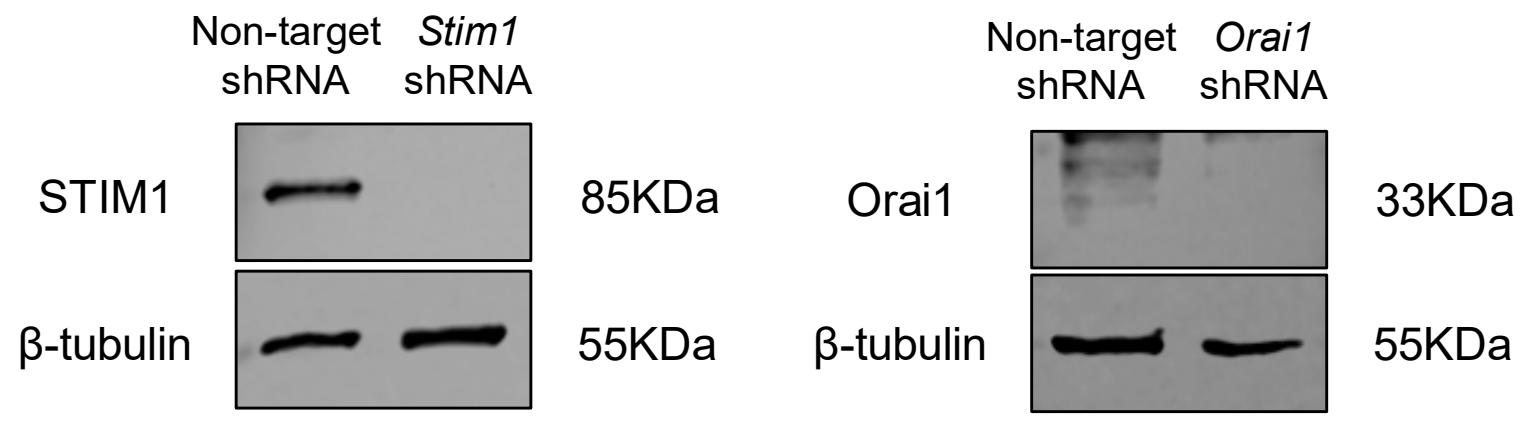

Supplemental Figure 8. Representative Western blots of STIM1 and Orai1 in mouse ASM cells expressing non-targeting, *Stim1*, or *Orai1* shRNA.

Representative Western blot showing STIM1 and Orai1 protein levels in cultured mouse ASM cells transduced with non-targeting, *Stim1*, or *Orai1* shRNA (n=3 biological replicates).

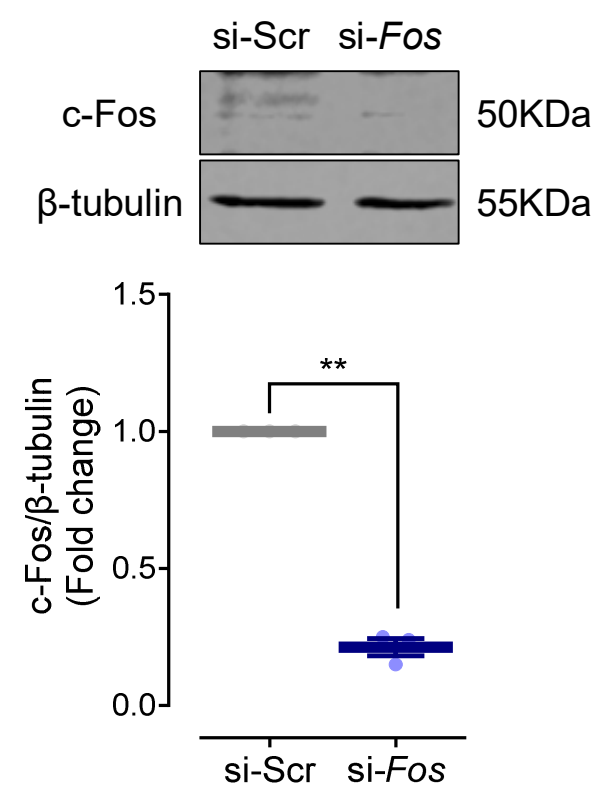

Supplemental Figure 9. Representative Western blots and quantification of c-Fos in mouse ASM cells transfected with siRNA.

Representative Western blot (top) and quantification (bottom) of c-Fos protein levels in whole-cell lysates from cultured mouse ASM cells transfected with scramble (si-Scr) or *Fos* (si-*Fos*) siRNA (n=3 biological replicates).

Data are presented as means ± SEM. Statistical analysis was performed by one-sample *t*-test. **p < 0.01.

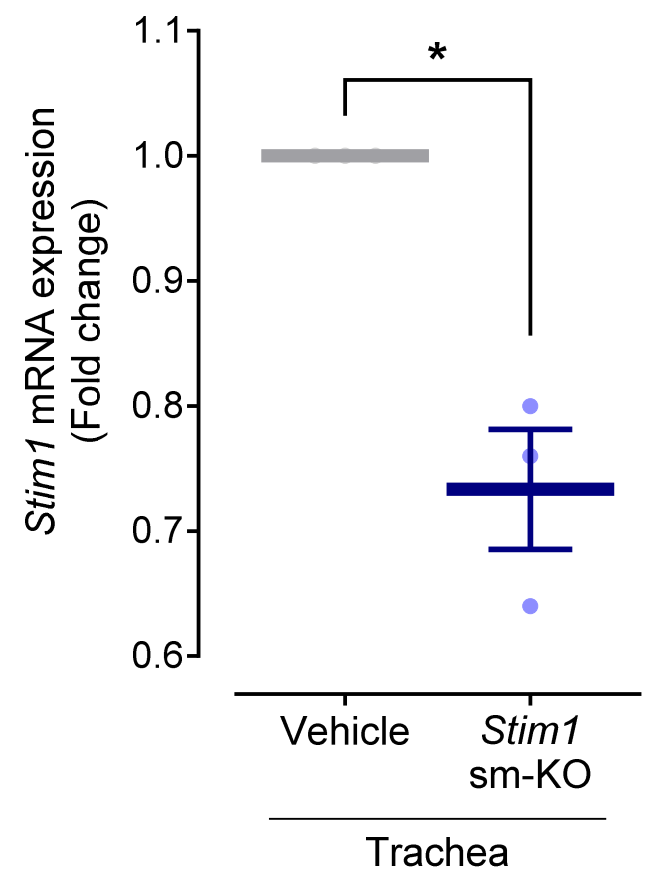

Supplemental Figure 10. Expression of *Stim1* in tracheal tissues from smooth muscle-specific *Stim1* knockout (*Stim1*-smKO) mice.

qPCR quantification of *Stim1* mRNA in tracheal tissues from *Myh11*^{CreERT2}:*Stim1*$^{fl/fl}$ mice treated with sunflower oil (vehicle) or tamoxifen (*Stim1*-smKO) (n=3 biological replicates). Data are presented as means ± SEM. Statistical analysis was performed by one-sample *t*-test. *$p < 0.05$.

Cohort study #1

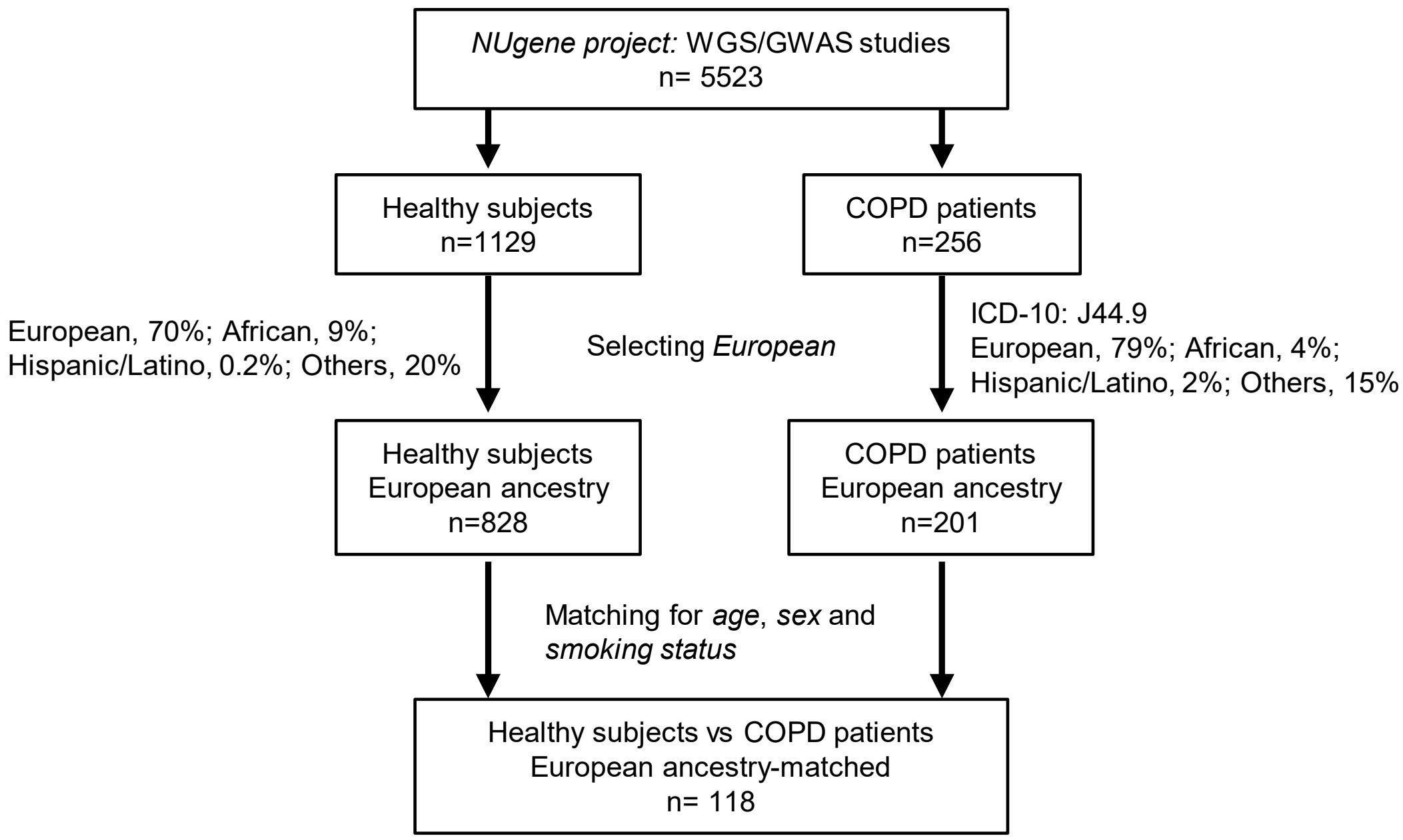

Cohort study #2

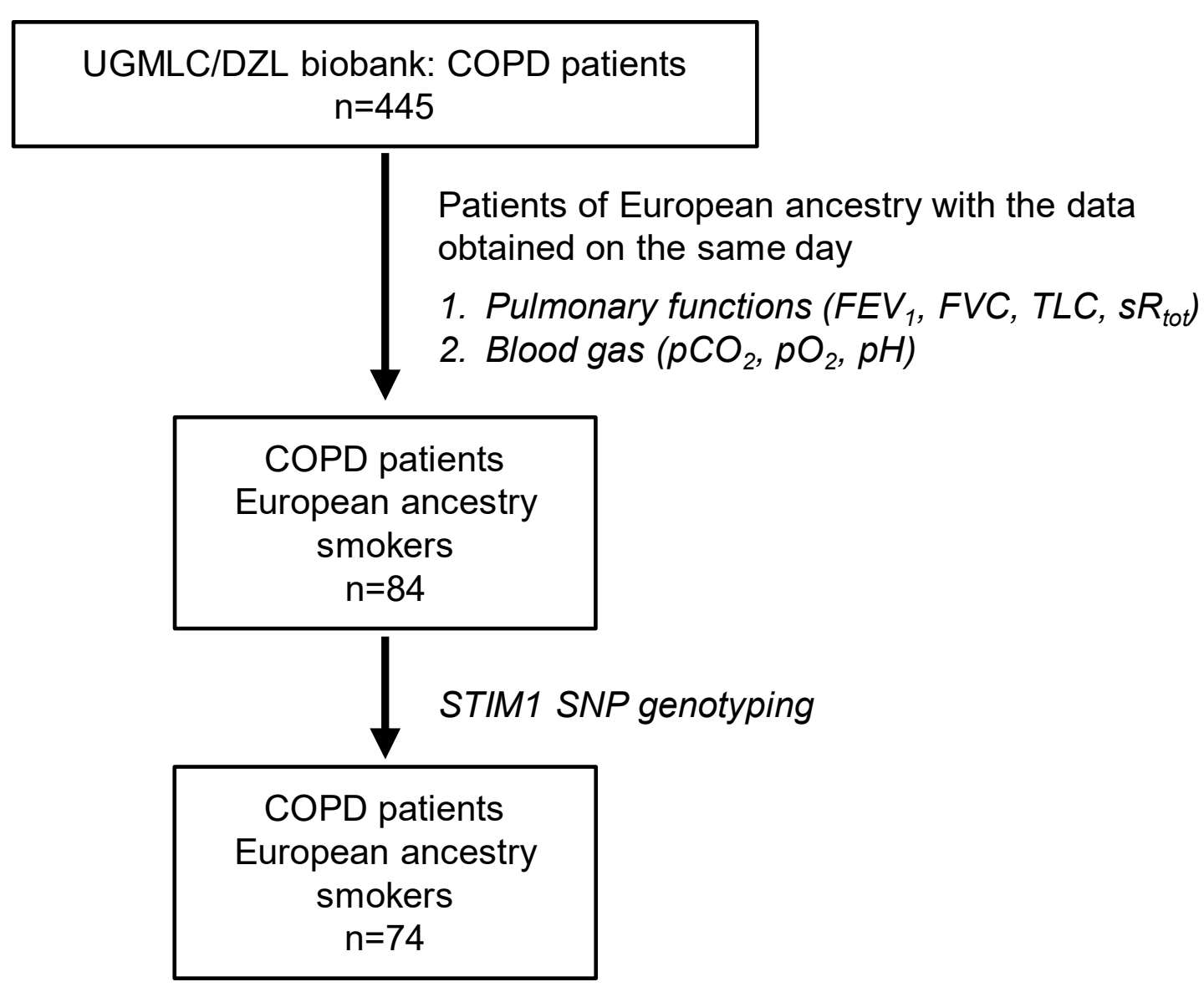

Supplemental Figure 11. The design of the cohort studies.

Supplemental Table 1. Characteristics of the study population in 828 healthy individuals of European ancestry

Demographic data

	Healthy subjects
No. of participants	828
Sex, Men/Women	170/658
Smoking status, Never/Ever	480/348
Age (yr)	47 (19-83)
BMI (kg/m^2)	28 (14-70)

STIM1 variants genotypes and allele frequency

		All European (n=828)	Sex		Smoking status		P value[A]
			Male (n=170)	Female (n=658)	Never (n=480)	Ever (n=348)	
rs1561786	AA	625 (75.5%)	127 (74.7%)	498 (75.7%)	358 (74.6%)	267 (76.7%)	
	AG	188 (22.7%)	39 (22.9%)	149 (22.6%)	112 (23.2%)	76 (21.8%)	
	GG	15 (1.8%)	4 (2.4%)	11 (1.7%)	10 (2.1%)	5 (1.4%)	
	A	1438 (86.8%)	293 (86.2%)	1145 (87.0%)	828 (86.3%)	610 (87.6%)	
	G	218 (13.2%)	47 (13.8%)	171 (13.0%)	132 (13.8%)	86 (12.4%)	0.5<
rs3794050	GG	633 (76.4%)	128 (75.3%)	505 (76.7%)	367 (76.5%)	266 (76.4%)	
	GA	185 (22.3%)	39 (22.9%)	146 (22.2%)	106 (22.1%)	79 (22.7%)	
	AA	10 (1.2%)	3 (1.8%)	7 (1.1%)	7 (1.5%)	3 (0.9%)	
	G	1451 (87.6%)	295 (86.8%)	1156 (87.8%)	840 (87.5%)	611 (87.8%)	
	A	205 (12.4%)	45 (13.2%)	160 (12.2%)	120 (12.5%)	85 (12.2%)	0.6<

Data are presented as mean (range) in Demographic data.
[A]Chi-square tests were performed to compare each subgroup against the All European.

Supplemental Table 2. Association between *STIM1* variants and COPD severity in Cohort #2

rs1561786				
	Normocapnic patients		Hypercapnic patients	
	AG	GG	AG	GG
Mild-to-Moderate (GOLD I–II)	2 (40%)	7 (33%)	1 (11%)	3 (7%)
Severe-to-Very severe (GOLD III-IV)	3 (60%)	14 (67%)	8 (89%)	36 (92%)
rs3794050				
	Normocapnic patients		Hypercapnic patients	
	GA	AA	GA	AA
Mild-to-Moderate (GOLD I–II)	2 (40%)	7 (33%)	1 (11%)	3 (7%)
Severe-to-Very severe (GOLD III-IV)	3 (60%)	14 (67%)	8 (89%)	36 (92%)

GOLD, Global Initiative for Obstructive Lung Disease.
