# Supplementary material for: Hypercapnia enhances airway smooth muscle contractility via STIM1-dependent Ca2+ signaling
Source: JCI Insight. 2026 Jul 16;11(17):e202475. doi: 10.1172/jci.insight.202475 (PMC13564089; doi:10.1172/jci.insight.202475)
Supplement: Supplementary file 2 [file jciinsight-11-202475-s119.pdf]

D

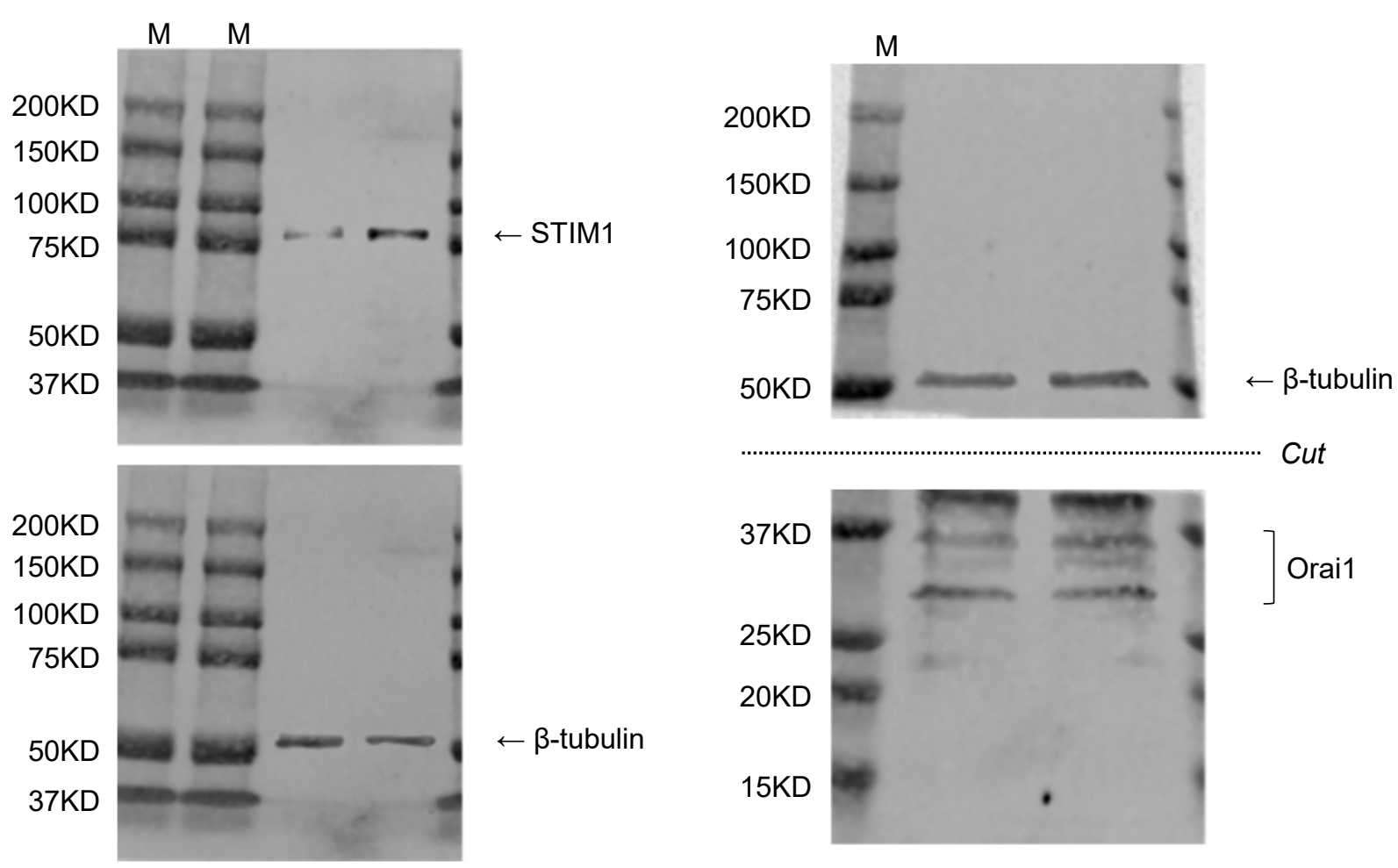

M, Protein marker

Figure 1

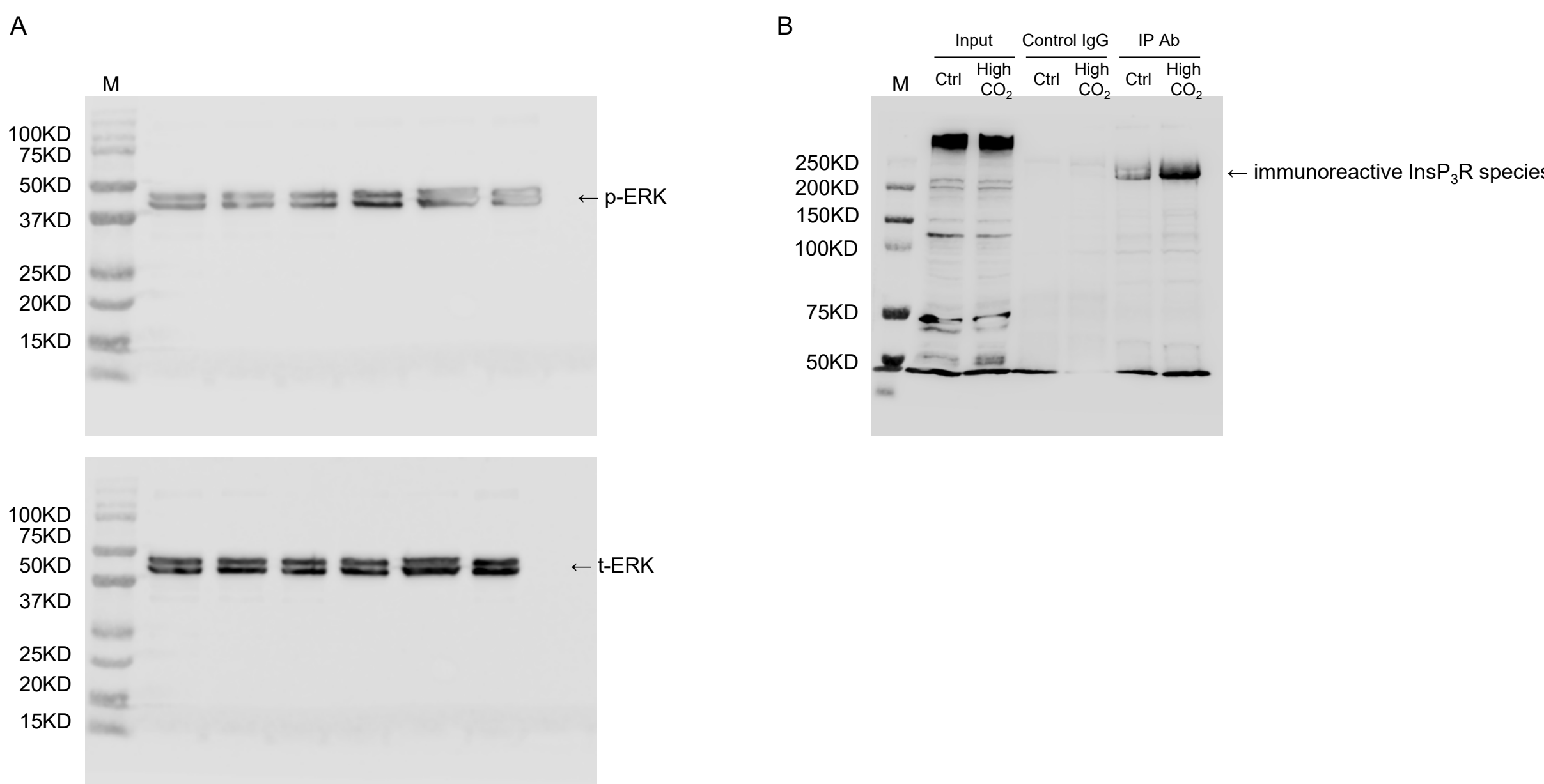

M, Protein marker

Figure 2

B C

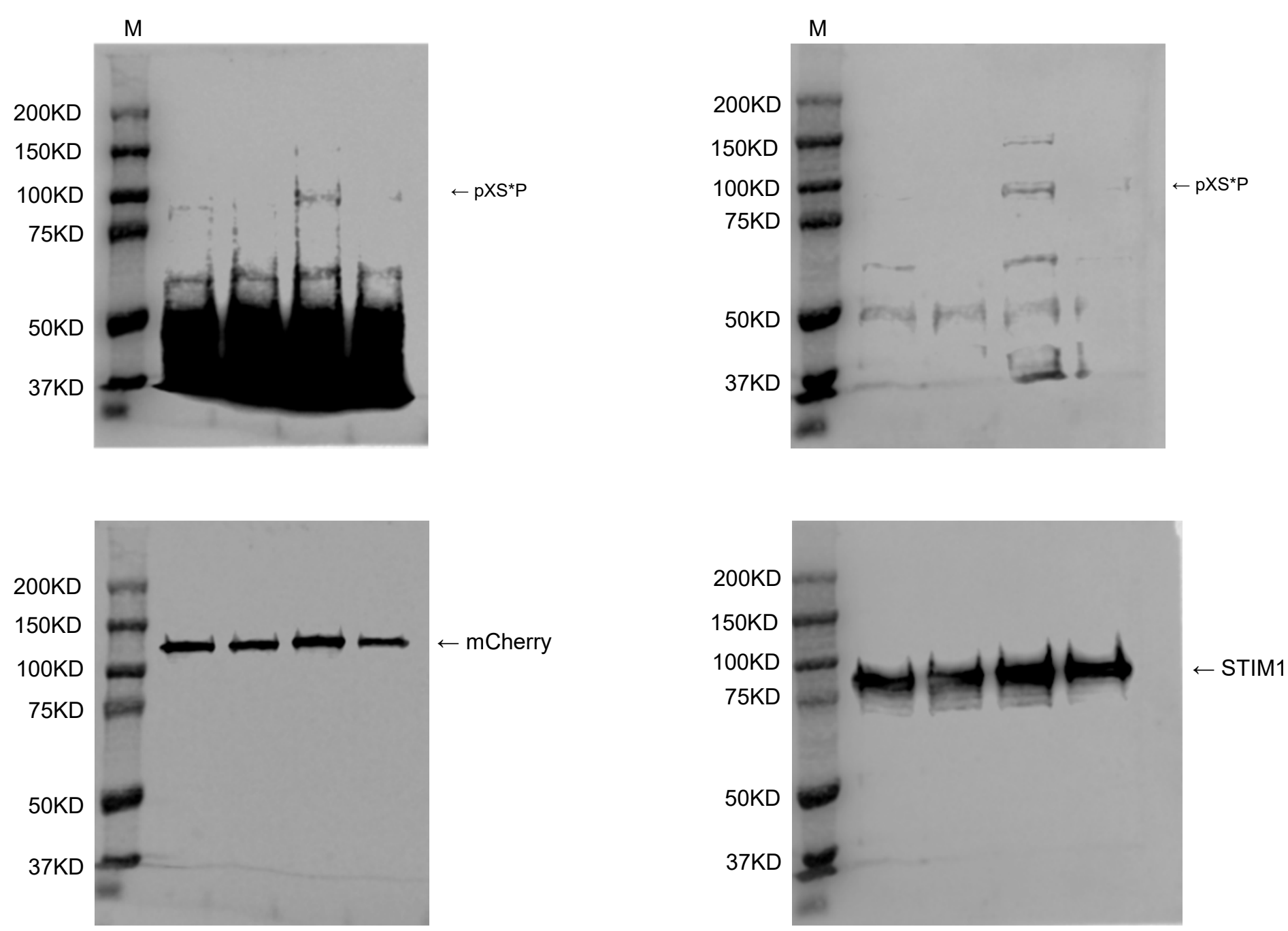

M, Protein marker

Figure 3

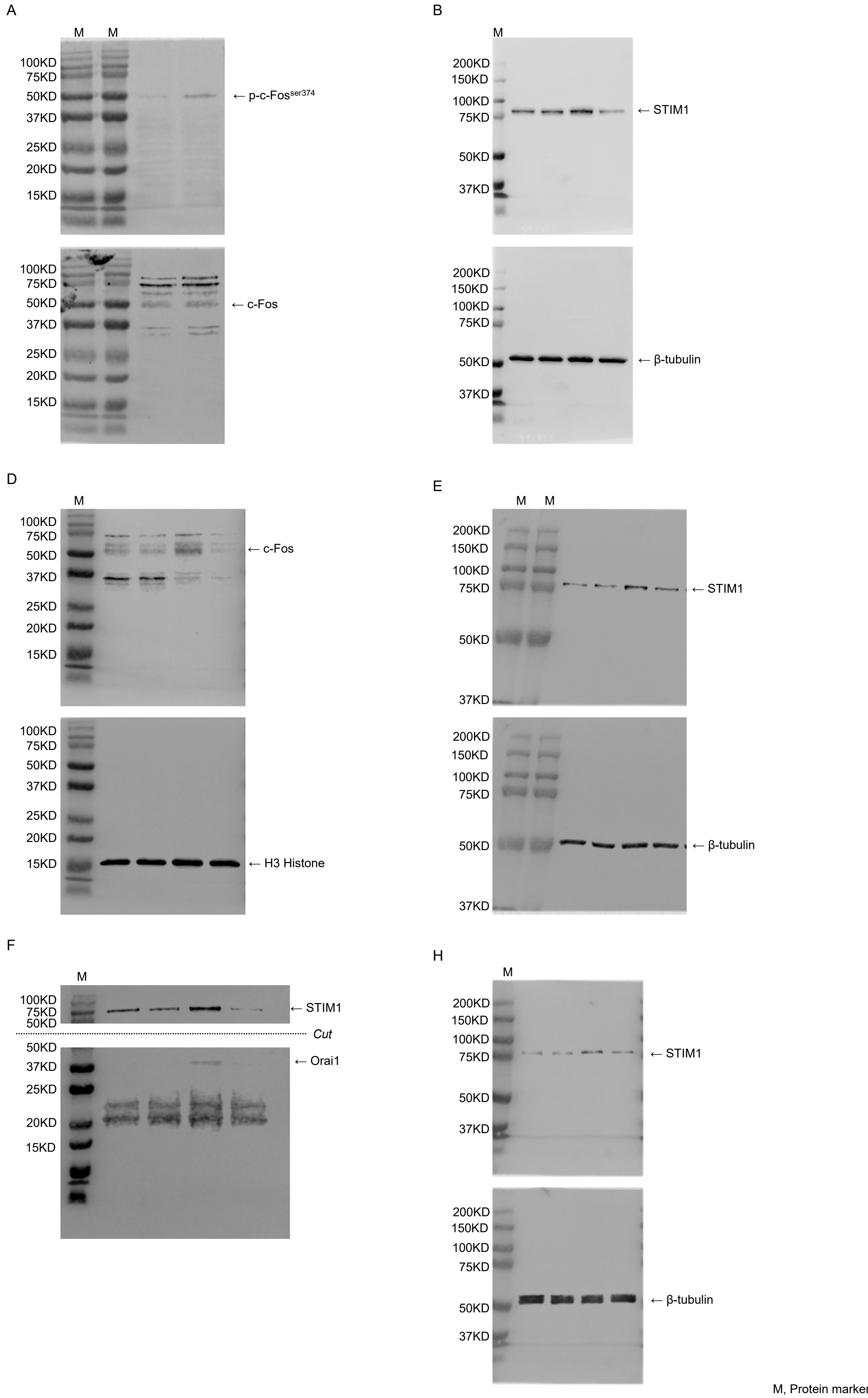
A
M M
100KD
75KD
50KD
37KD
25KD
20KD
15KD
← p-c-Fos ser374
← c-Fos
B
M
200KD
150KD
100KD
75KD
50KD
37KD
← STIM1
← β-tubulin
D
M
← c-Fos
← H3 Histone
E
M M
← STIM1
← β-tubulin
F
M
← STIM1
Cut
← Orai1
H
M
← STIM1
← β-tubulin
M, Protein marker

Figure 4

B

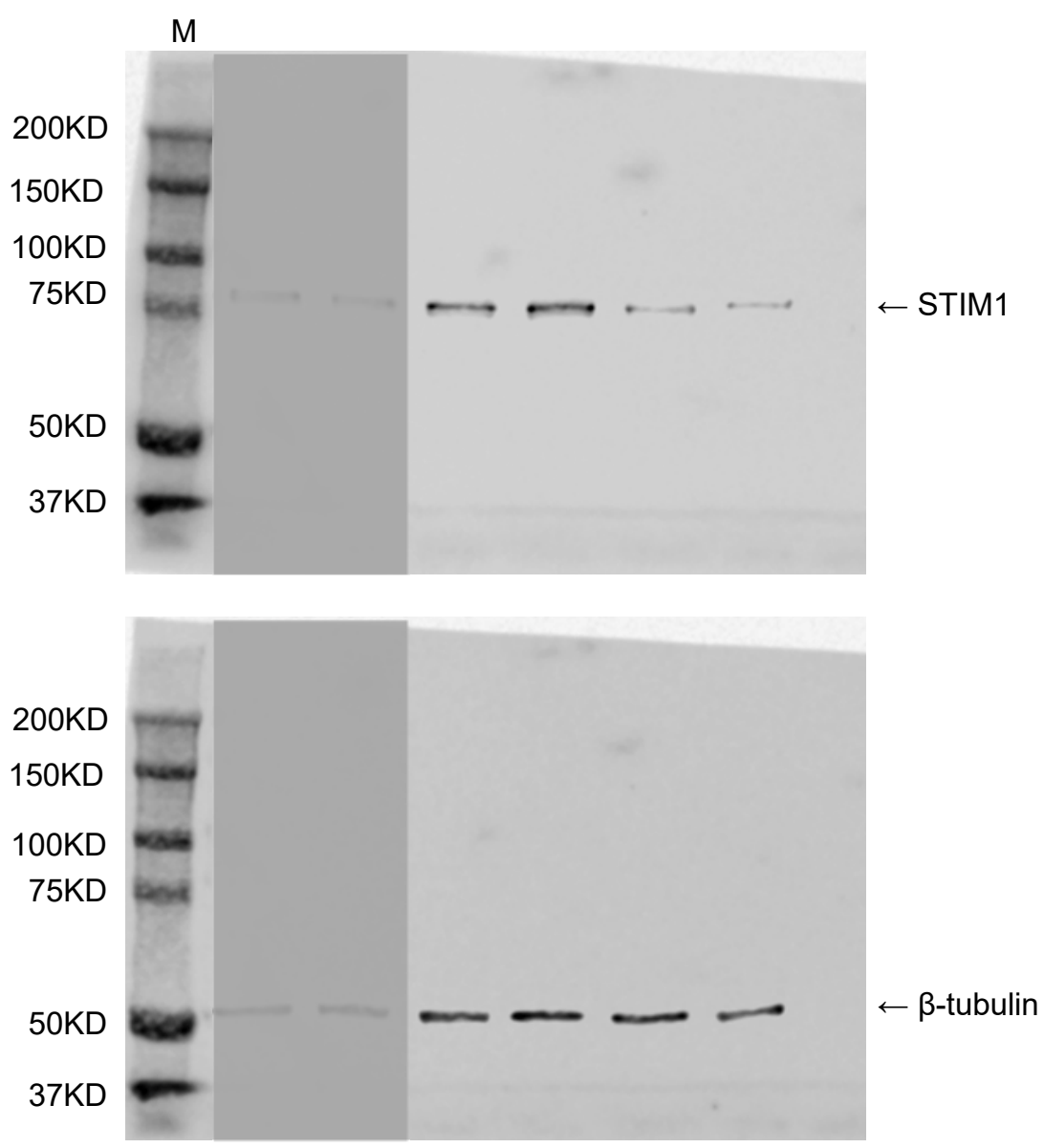

D

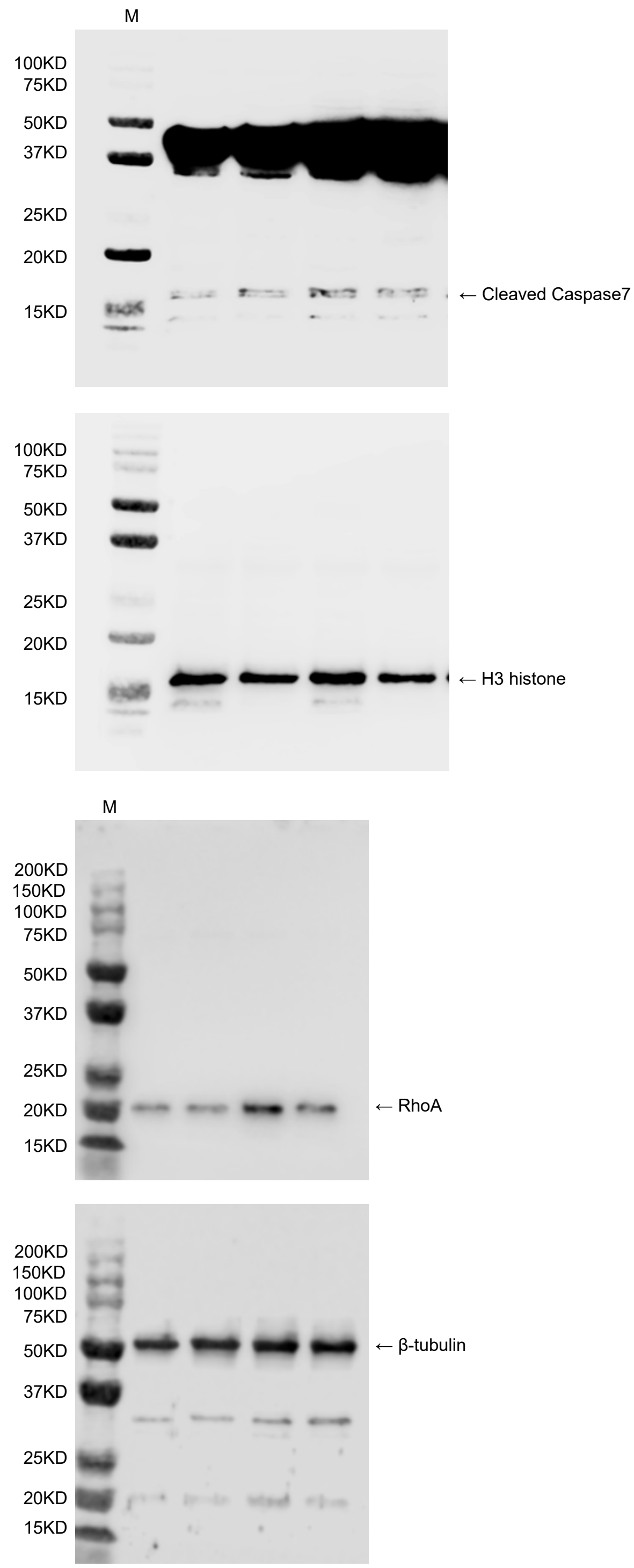

M, Protein marker

Figure 5

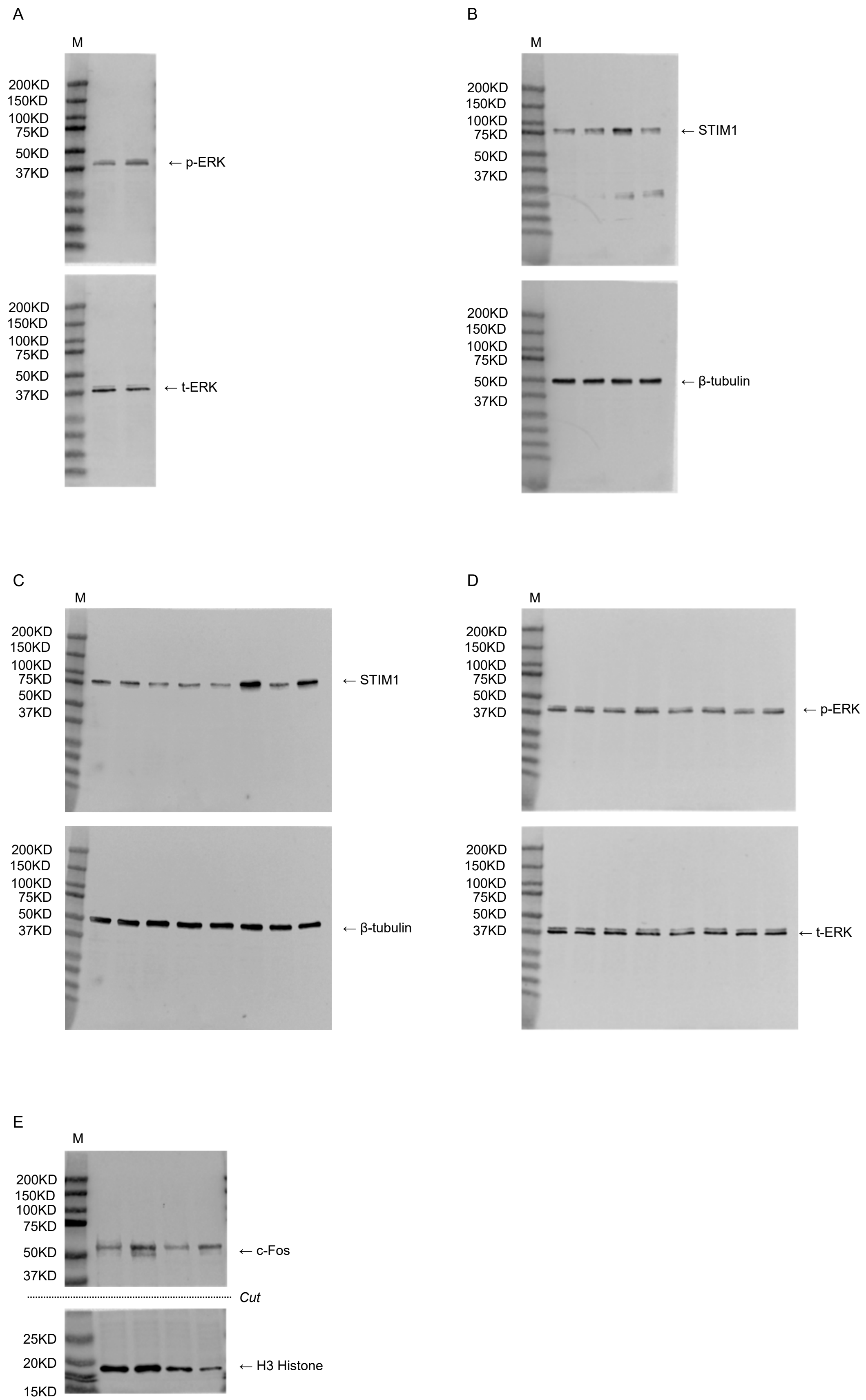

M, Protein marker

Figure 6

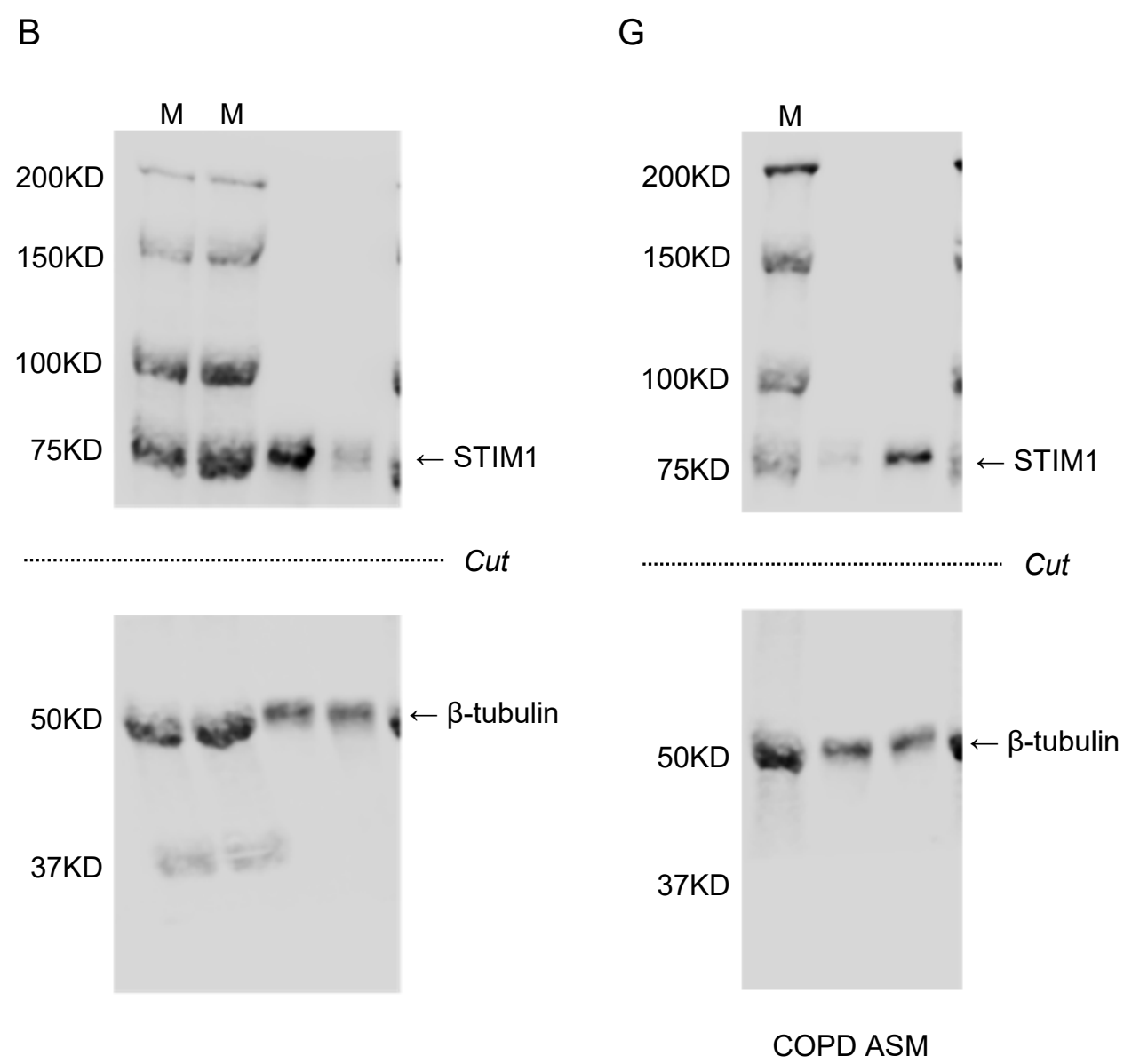

M, Protein marker

Figure 7

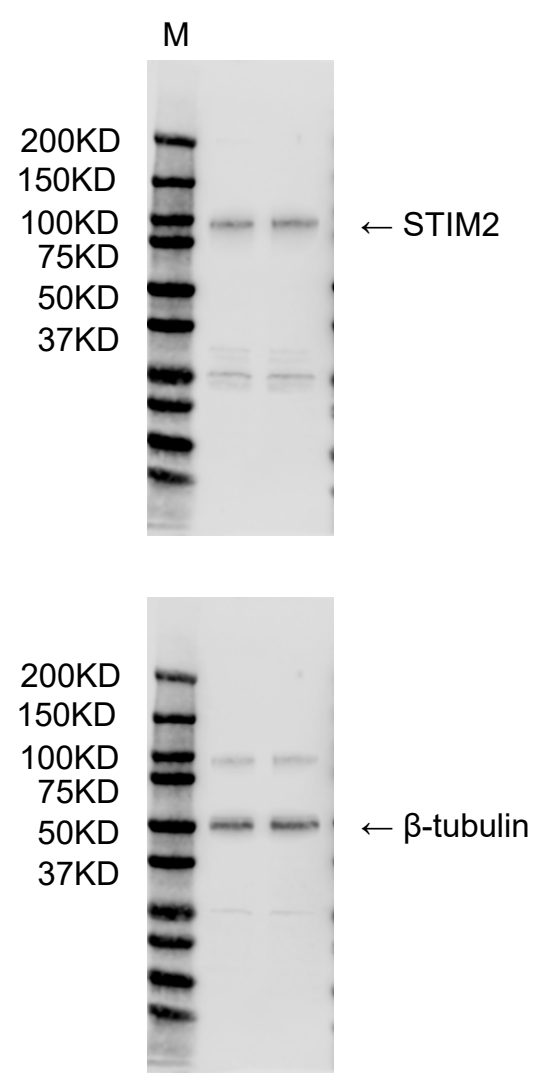

M, Protein marker

Supplemental Figure 3

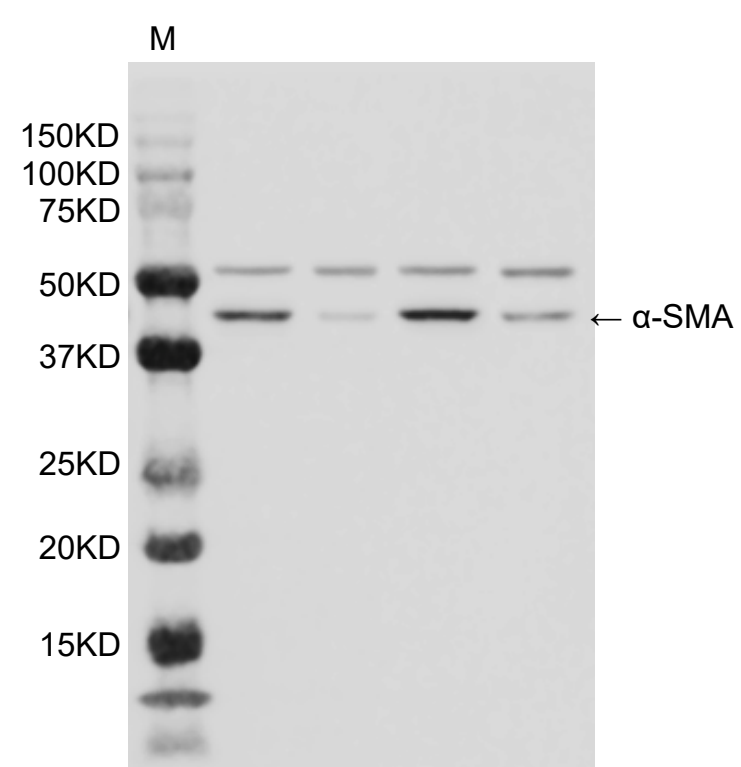

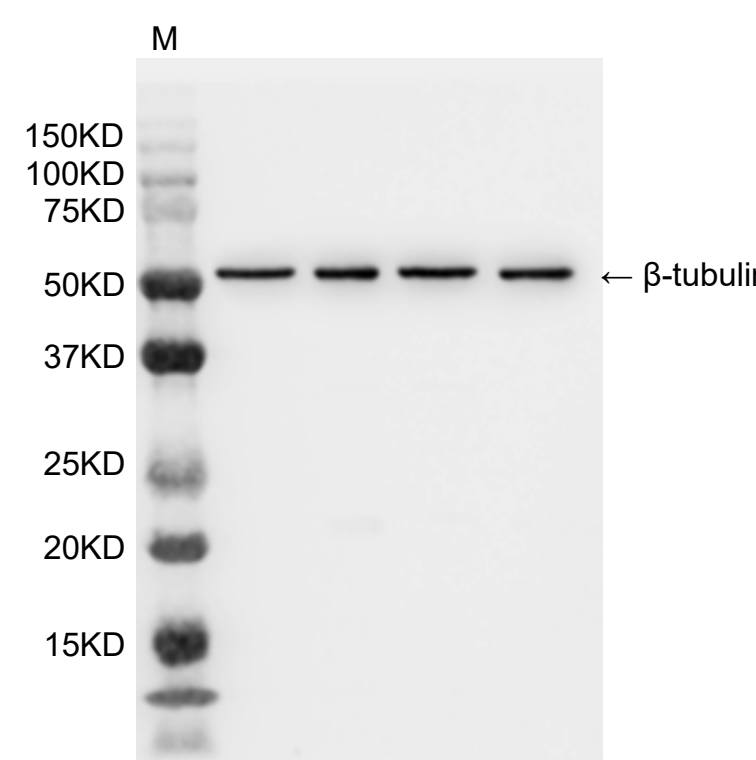

M, Protein marker

Supplemental Figure 4

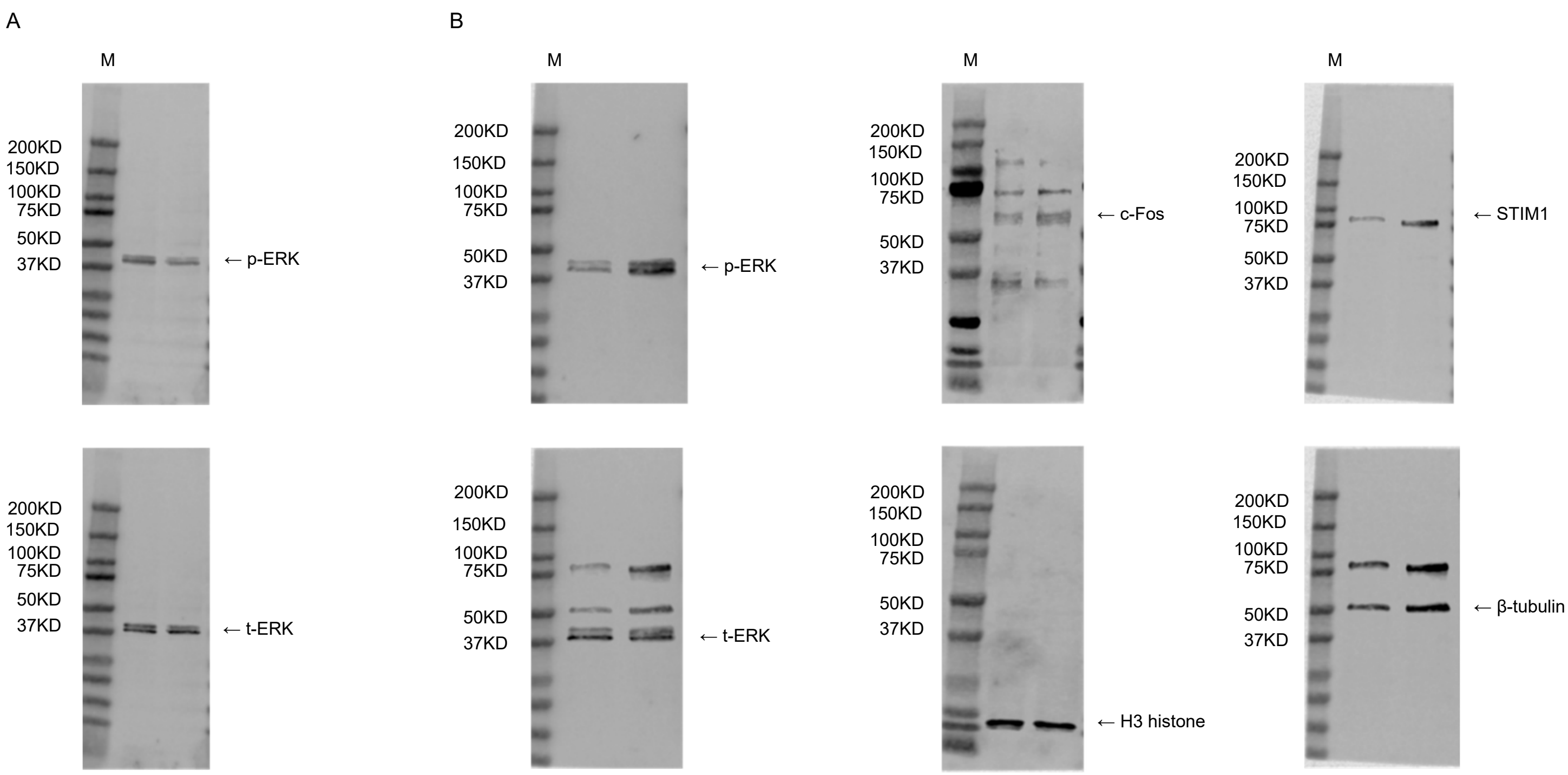

M, Protein marker

Supplemental Figure 5

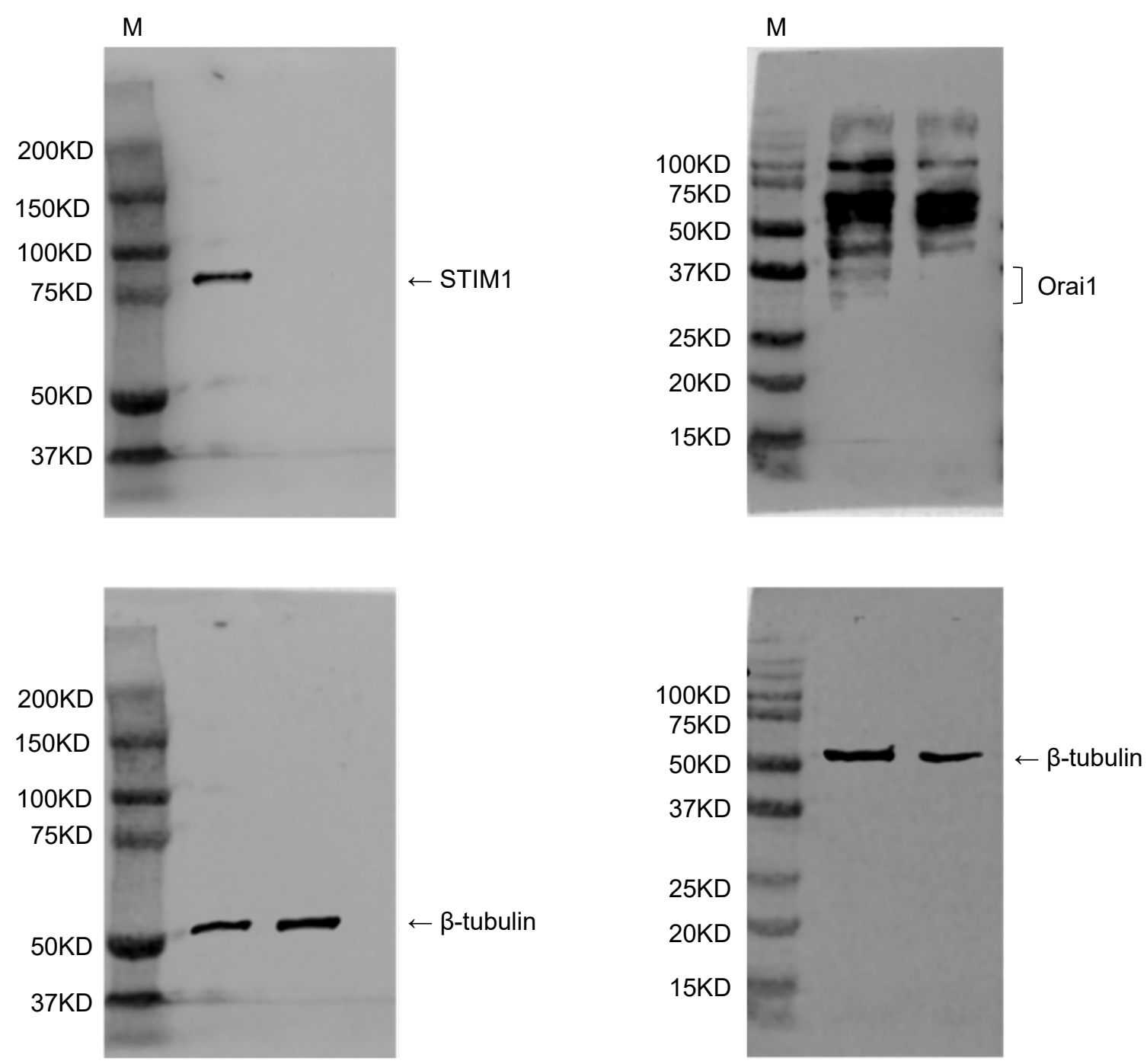

M, Protein marker

Supplemental Figure 8

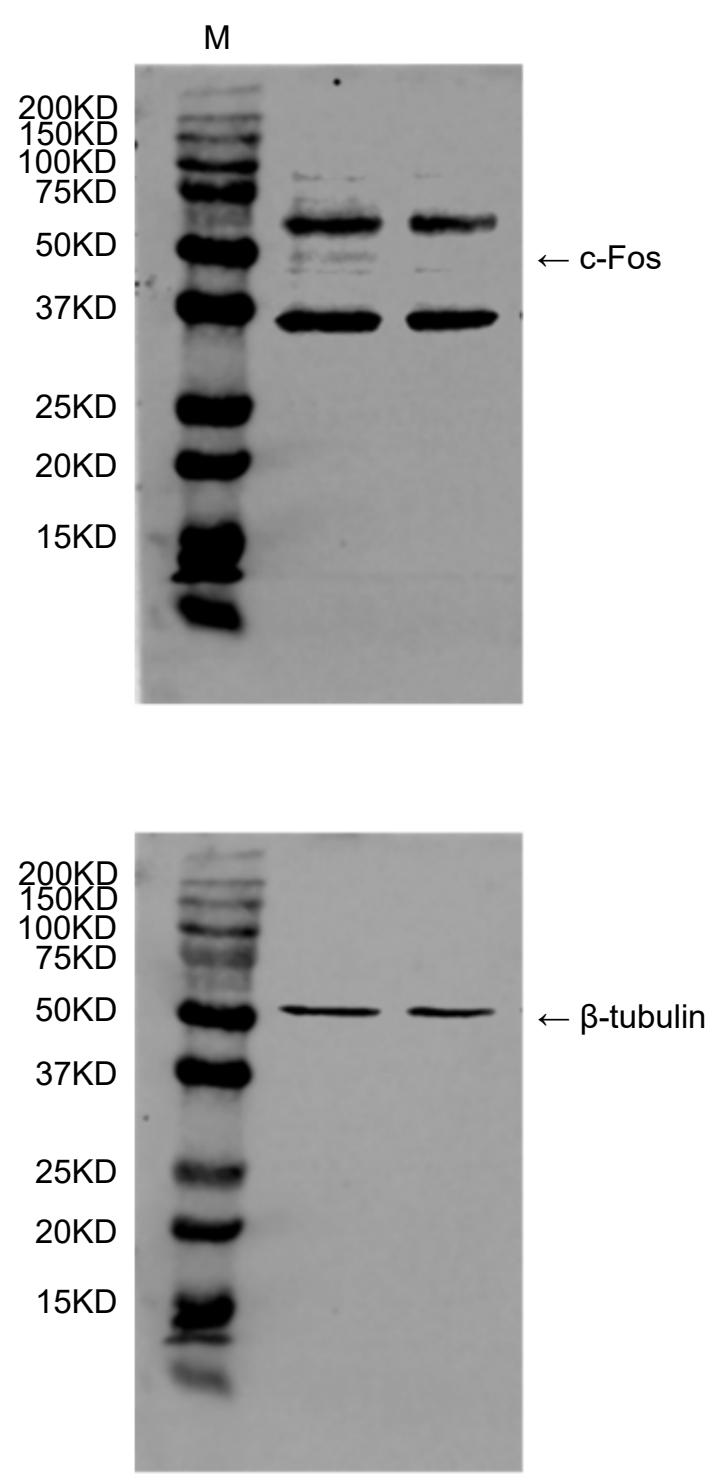

M, Protein marker

Supplemental Figure 9
